# In Vitro and In Silico Pharmacological Study of Three Combined Lamiaceae Essential Oils: Cytotoxicity and Antiviral Potential

**DOI:** 10.3390/molecules30214182

**Published:** 2025-10-25

**Authors:** Aicha Khemili, Djamel Bensizerara, Haroun Chenchouni, Tomasz Gębarowski, Barbara Bażanów, Taha Menasria, Anna Tomańska, Aleksandra Chwirot, Antoni Szumny

**Affiliations:** 1 Department of Molecular and Cellular Biology, Faculty of Nature and Life Sciences, University of Abbes Laghrour, Khenchela 40000, Algeria; 2 Laboratory of Biotechnology, Water, Environment and Health, University of Abbes Laghrour, Khenchela 40000, Algeria; dbensizerara@yahoo.fr; 3 Department of Agronomy, Faculty of Nature and Life Sciences, University of Abbes Laghrour, Khenchela 40000, Algeria; 4 Laboratory of Natural Resources and Management of Sensitive Environments ‘LRNAMS’, University of Oum El Bouaghi, Oum El Bouaghi 04000, Algeria; 5 Department of Forest Management, Higher National School of Forests, Khenchela 40000, Algeria; 6 Department of Biostructure and Animal Physiology, Faculty of Veterinary Medicine, Wrocław University of Environmental and Life Sciences, Kożuchowska St. 1, 51-631 Wrocław, Poland; tomasz.gebarowski@upwr.edu.pl (T.G.); anna.tomanska@upwr.edu.pl (A.T.); 7 Department of Pathology, Division of Microbiology, Faculty of Veterinary Medicine, Wrocław University of Environmental and Life Sciences, 31 C. K. Norwida St., 50-573 Wrocław, Poland; barbara.bazanow@upwr.edu.pl (B.B.); aleksandra.pogorzelska@upwr.edu.pl (A.C.); 8 Department of Microbiology and Biochemistry, Faculty of Nature and Life Sciences, University of Batna 2, Batna 05078, Algeria; t.menasria@univ-batna2.dz; 9 Department of Food Chemistry and Biocatalysis, Wrocław University of Environmental and Life Sciences, Norwida 25, 50-375 Wrocław, Poland

**Keywords:** essential oil, *Rosmarinus officinalis*, *Salvia officinalis*, *Mentha × piperita*, anticancer activity, antiviral activity, combination

## Abstract

This study investigated the in vitro and in silico anticancer and antiviral potential of three Lamiaceae essential oils (EOs), *Rosmarinus officinalis* (REO), *Salvia officinalis* (SEO), and *Mentha × piperita* (MEO). The essays included both Eos tested individually and in combination. Cytotoxicity was assessed in normal dermal fibroblast (NHDF), breast (MCF7), lung (A549), and colorectal (LoVo) cell lines. Antiviral activity was evaluated against herpes simplex virus type 1 (HSV-1) and adenovirus type-5 (AdV-5). Major identified compounds were subjected to in silico analysis against selected cancer- and virus-related protein targets. None of the EOs or their combinations showed cytotoxicity toward NHDF cells. REO exhibited significant anticancer activity against MCF7 and A549 cells, while SEO displayed the greatest antiproliferative effect on MCF7 cells. MEO showed moderate activity against MCF7 cells and weak activity against A549 cells. All EOs and combinations showed limited efficacy against LoVo cells. Combined EOs were more effective against A549 cells, showing synergism for REO combinations, whereas lower activity was noted against MCF7 cells, where the MEO + SEO combination exhibited an antagonistic effect. All EOs and their combinations effectively reduced HSV-1 and AdV-5 titers. In silico results confirmed the binding affinities between the major EO compounds and selected protein targets, supporting their potential as complementary therapeutic agents.

## 1. Introduction

The exploration of medicinal plants has a long and rich history, with interest in their chemical constituents intensifying in the 18th and 19th centuries [1]. Early phytochemical research focused on isolating specific bioactive compounds believed to be responsible for the therapeutic effects, giving rise to substances such as morphine, quinine, and caffeine [1,2]. While this reductionist approach helped shape modern pharmacology, it gradually provided a more holistic understanding of plant-based therapies [1]. Advances in analytical techniques throughout the 20th century have highlighted the value of complex natural mixtures, shifting the focus toward rational phytotherapy and the synergistic interplay of multiple compounds [1,3]. Today, rising costs and ecological concerns surrounding single-compound drug development have further emphasized the importance of natural formulations [3]. Notably, pure substances, when administered at concentrations equivalent to those found in natural mixtures, often fail to achieve the same qualitative and quantitative pharmacological effects [3]. Studies now show that plant extracts often exhibit greater efficacy than isolated compounds due to their multi-target, synergistic actions [3,4]. These interactions may enhance therapeutic outcomes (synergism), moderate toxicity (antagonism), or broaden the spectrum of biological activity (additive effects) [5].

Among the most versatile and longstanding plant preparations are essential oils (EOs), which are central to both traditional remedies and modern medicine, and are valued for their diverse biological effects [6]. Their versatility and alignment with sustainable and integrative health approaches have renewed scientific interest in their full potential [7]. Additionally, EOs can be administered through various routes, including oral, rectal, and vaginal, alongside more commonly known methods such as inhalation and topical application [8]. Chemically, EOs are complex mixtures composed primarily of terpenes and phenylpropanoids, which are largely responsible for their biological activities [9]. While major constituents often define the biological potential of an EO, minor components can also exert meaningful effects [5]. Their interactions underline the importance of both quantitative and qualitative chemical analyses [9]. EO constituents can interact with each other and with cellular targets such as proteins and receptors, contributing to their wide-ranging pharmacological effects [4,9].

Despite these promising features, the interactions between EO components as well as among different EOs, whether synergistic, additive, or antagonistic, remain underexplored, particularly in the context of complex diseases such as cancer and viral infections [10]. These conditions continue to pose major global health challenges, contributing significantly to morbidity, mortality, and economic strain [10]. Cancer affects millions of people worldwide, and its rising incidence is fueled by aging populations, lifestyle changes, and environmental factors [3]. Viral infections, ranging from endemic illnesses to devastating pandemics, continue to disrupt societies worldwide [11]. The impact of these diseases extends beyond health, placing severe pressure on economies and health care systems. Addressing such challenges demands a unified international effort to develop innovative, accessible, and sustainable therapeutic strategies [7,12].

This study investigated the anticancer and antiviral properties of three EOs: *Salvia rosmarinus* Spenn. (REO), *Mentha × piperita* L. (MEO), and *Salvia officinalis* L. (SEO). These plants were selected based on their traditional use and relevance in the pharmaceutical and food industries [13]. We aimed to assess potential synergistic effects by (i) evaluating their cytotoxicity against normal dermal fibroblasts and three human cancer cell lines, and (ii) testing their antiviral activity against human herpes simplex virus type 1 (HSV-1) and human adenovirus type 5 (AdV5), both individually and in combination.

## 2. Results

### 2.1. Chemical Profiling

The aromatic profiles of the aerial parts of REO, MEO, and SEO, obtained using gas chromatography-mass spectroscopy (GC-MS) analysis, as well as the EO extraction yield, are summarized in Table 1. The yield of EOs from the aerial parts ranged from 1.1 to 1.8%. REO had the highest yield (1.8%), whereas the MEO and SEO samples yielded 1.1% and 1.4%, respectively. In the REO sample, a total of 61 compounds were identified, accounting for 98.92% of the total oil composition. The primary compound was camphor (23.52%), followed by α-pinene (21.20%), camphene (18.94%), eucalyptol (5.02%), β-caryophyllene (4.17%), limonene (3.27%), β-pinene (3.28%), *p*-cymene (2.66%), and δ-cadinene (2.38%). The REO compounds were categorized as hydrocarbon monoterpenes (53.35%), oxygenated monoterpenes (32.52%), hydrocarbon sesquiterpenes (12.39%), and oxygenated sesquiterpenes with small amount (0.62%).

In MEO, there was a total of 99.20%, represented by 47 compounds. The main components were γ-terpineol, dihydro- (43.50%), and menthone (25.97%), both of which belong to the class of oxygenated monoterpenes, with an overall contribution of 93.14%. Other significant compounds included isomenthone (9.61%), menthyl acetate (5.98%), and eucalyptol (4.99%). Hydrocarbon monoterpenes were present at 4.27%, whereas hydrocarbon and oxygenated sesquiterpenes appeared only as traces at 1.31% and 0.12%, respectively.

The SEO sample examined in this study contained 42 compounds, representing 96.14% of the total volatile compounds, with eucalyptol (23.57%), α-thujone (22.02%), and camphor (20.5%) being the most abundant. Oxygenated monoterpenes were the predominant family, representing 73.81%. Notably, β-thujone (4.37%) was also detected. Hydrocarbon monoterpenes comprised 13.98%, with camphene (4.17%), β-pinene (3.1%), β-myrcene (2.2%), and α-pinene (2.13%) being the major components. Hydrocarbon sesquiterpenes accounted for 5.73%, with isocaryophyllene (3.21%) as the predominant compound, whereas hydrocarbon sesquiterpenes accounted for 1.93%. Additionally, SEO contained a diterpene (0.15%).

### 2.2. In Vitro Anticancer Activity

**Figure 1:**
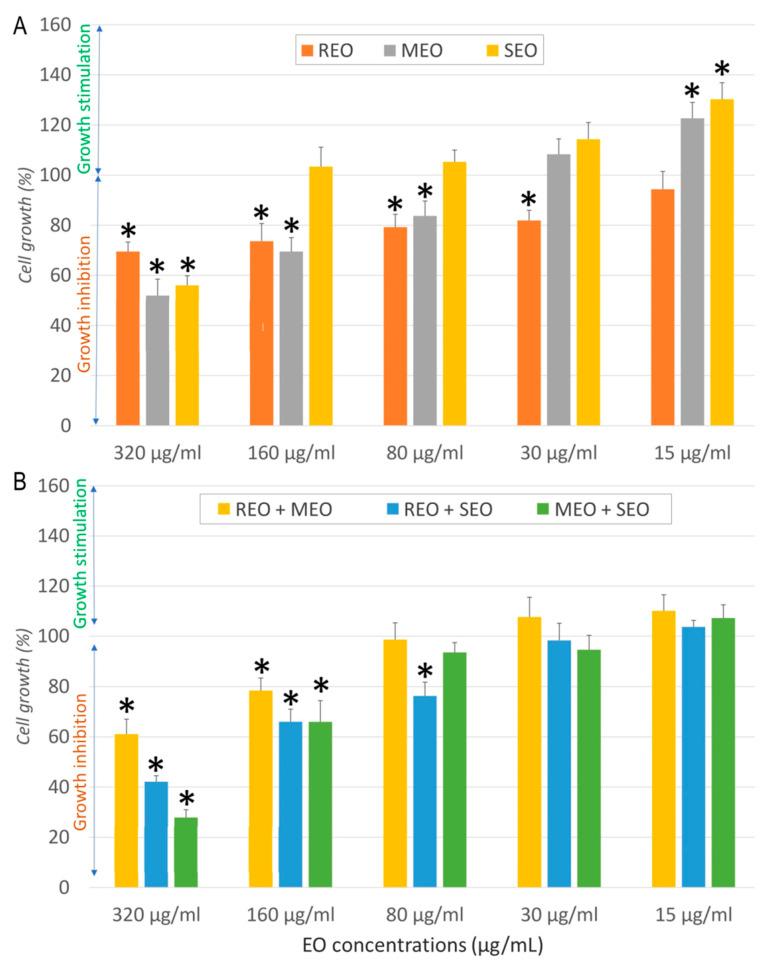
Effect of *Salvia rosmarinus*, *Mentha × piperita*, and *Salvia officinalis* essential oils individually (**A**) and in combination (**B**) on NHDF cell growth. *: indicates a significant difference (*p* < 0.05) compared to the negative control following Tukey’s Post Hoc test (HSD).

**Figure 2:**
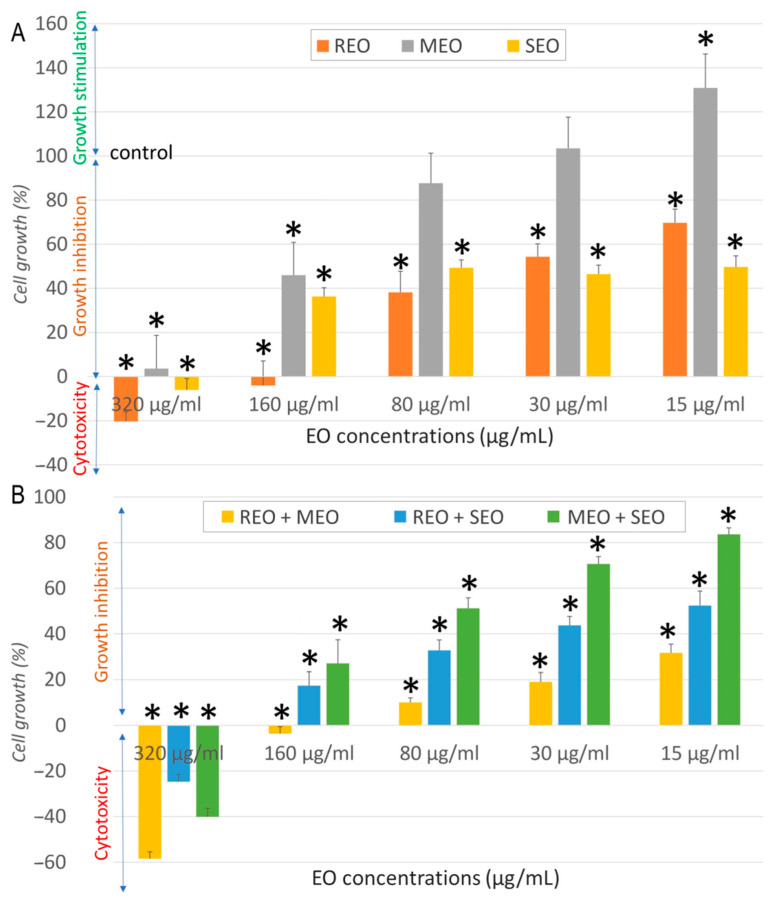
Cytotoxicity effect of *S. rosmarinus*, *M. × piperita,* and *S. officinalis* essential oils individually (**A**) and in combination (**B**) against A549 cells. *: indicates a significant difference (*p* < 0.05) compared to the negative control following Tukey’s Post Hoc test (HSD).

**Figure 3:**
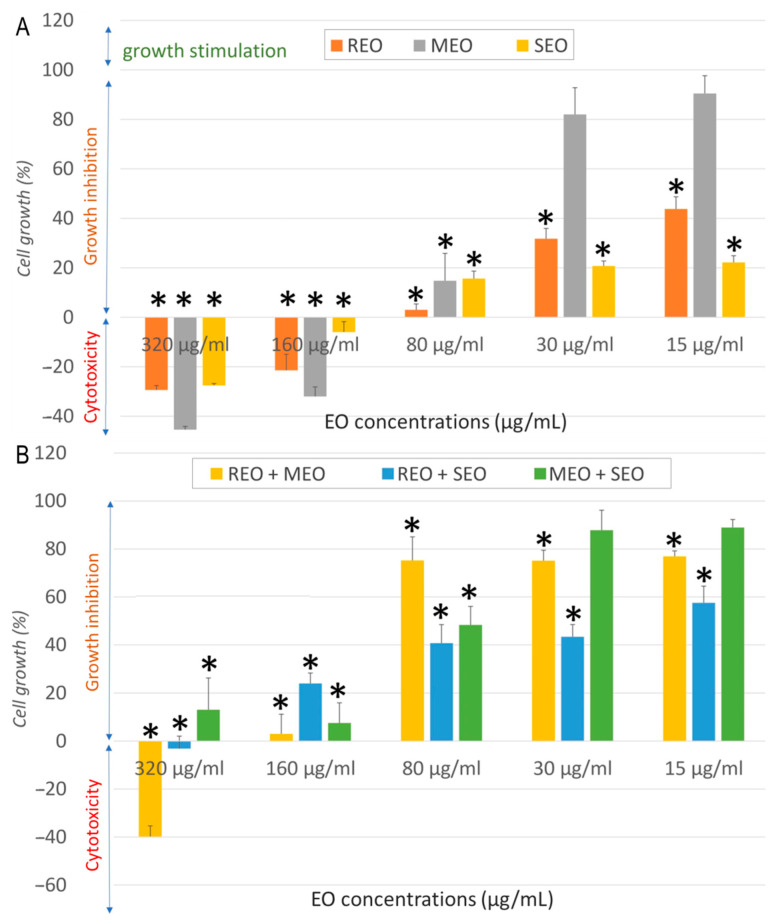
Cytotoxicity effect of *S. rosmarinus*, *M. × piperita*, and *S. officinalis* essential oils individually (**A**) and in combination (**B**) against MCF7 cells. *: indicates a significant difference (*p* < 0.05) compared to the negative control following Tukey’s Post Hoc test (HSD).

**Figure 4:**
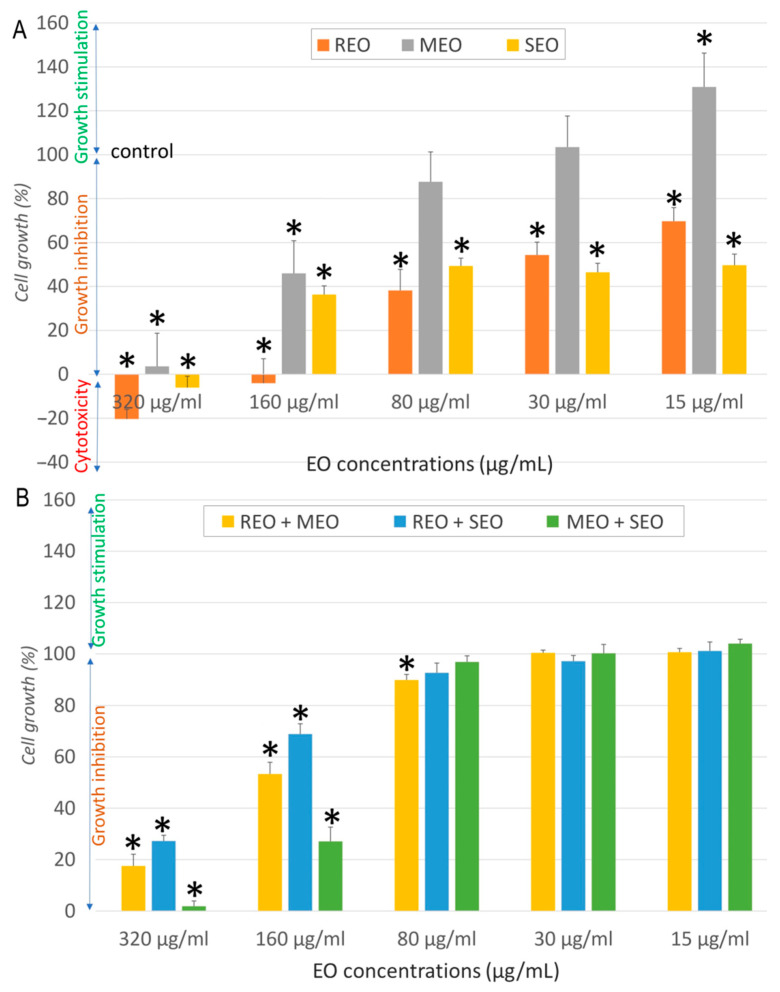
Cytotoxicity effect of *S. rosmarinus*, *M. × piperita*, and *S. officinalis* essential oils individually (**A**) and in combination (**B**) against LoVo cells. *: indicates a significant difference (*p* < 0.05) compared to the negative control following Tukey Post Hoc test (HSD).

The cytotoxic and antiproliferative effects of REO, MEO, SEO, and their combinations on normal human dermal fibroblast (NHDF), human breast cancer cell line (MCF7), human lung cancer cell line (A549), and human colorectal cancer cell line (LoVo) at various concentrations (15–320 µg/mL) are illustrated in Figure 1, Figure 2, Figure 3 and Figure 4. In NHDF cells (Figure 1), all EOs and their combinations showed dose-dependent effects. At the highest concentration (320 µg/mL), all three EOs moderately inhibited cell growth (Figure 1A). Compared to the negative control, a significant difference (*p* < 0.05) was observed for REO at 320, 160, 80, and 30 µg/mL, for MEO at 320, 160, 80, and 15 µg/mL, and for SEO at 320 and 15 µg/mL. Whereas their combinations produced stronger inhibitory effects (Figure 1B). Compared to the negative control, a significant difference (*p* < 0.05) was observed for all combinations at 320 and 160 µg/mL, and for REO + SEO at 80 µg/mL. At lower concentrations, most samples showed minimal or stimulatory effects, indicating low cytotoxicity to normal cells (Figure 1).

As shown in Figure 2A, all individual EOs displayed marked cytotoxicity at 320 µg/mL, with REO and SEO reducing A549 cell growth below 0%, indicating cell death. MEO was comparatively less cytotoxic than other EOs. A clear dose-dependent recovery of cell viability was observed at lower concentrations. Compared to the negative control, a significant difference (*p* < 0.05) was observed for all tested EOs at 320 and 160 µg/mL, for REO and SEO at 80, 30, and 15 µg/mL, and for MEO at 15 µg/mL. Combination treatments in Figure 2B showed enhanced cytotoxicity, with viability dropping below −20% at 320 µg/mL. Notably, even the lowest concentrations continued to show significant inhibition. Compared to the negative control, a significant difference (*p* < 0.05) was observed for all tested combinations at all concentrations.

As shown in Figure 3A, the treatment caused dose-dependent growth inhibition in MCF7 cells. All individual EOs showed marked cytotoxicity at the highest concentrations tested (320 and 160 µg/mL). MEO was the most cytotoxic at higher concentrations, whereas at lower doses (30 and 15 µg/mL), it strongly stimulated cell growth. Other oils exhibited milder responses. Compared to the negative control, a significant difference (*p* < 0.05) was observed for all tested EOs at 320, 160, and 80 µg/mL, and for REO and SEO at 30 and 15 µg/mL. When combined, EOs amplified both cytotoxic and stimulatory responses (Figure 3B). REO + MEO showed pronounced cytotoxicity at high doses, whereas MEO + SEO showed no proliferative effect, only slight decrease in cell growth. Compared to the negative control, a significant difference (*p* < 0.05) was observed for all combinations at 320, 160, and 80 µg/mL, and for REO-based combinations at 30 and 15 µg/mL.

Figure 4 indicates that none of the EOs and their combinations caused complete cytotoxicity in LoVo cells. At 320 and 160 µg/mL, MEO and the MEO + SEO combination showed significant growth inhibition, whereas the other samples had moderate effects. Compared to the negative control, a significant difference (*p* < 0.05) was observed at all concentrations of REO and SEO, and at 320, 160, and 15 µg/mL for MEO (Figure 4A). A significant difference (*p* < 0.05) compared to the negative control was also observed for all tested combinations at 320 and 160 µg/mL, and for REO + MEO at 80 µg/mL (Figure 4B).

For all treatments, as concentrations decreased, cell viability is not affected. These results reinforce the concentration- and combination-dependent nature of EO activity, with high-dose cytotoxicity and potential low-dose proliferative effects in different cell lines.

Table 2 summarizes the cytotoxicity data of the tested samples, including IC_50_ (50% inhibitory concentration), SI (selectivity index), and FIC (fractional inhibitory concentration) indices. The EOs and their combinations exhibited no cytotoxicity against NHDF cells, with IC_50_ values ranging from 223.6 to 537.7 µg/mL.

When tested individually, REO and SEO exhibited the strongest anticancer activity against MCF7 cells, with low IC_50_ values of 13.7 ± 2.6 and 9.7 ± 0.8 µg/mL, respectively. These results highlight the high selectivity of REO and SEO toward MCF7, with SI values of 39 and 33.6, respectively, suggesting a favorable therapeutic window for these EOs against breast cancer. REO also displayed considerable activity against A549 cells (IC_50_ = 32.3 ± 6.6 µg/mL, SI = 16.6), while SEO showed moderate anticancer activity on this cell line (IC_50_ = 80.5 ± 10.9 µg/mL, SI = 4).

Additionally, MEO exhibited moderate cytotoxicity against MCF7 cells (IC_50_ = 50.9 ± 15.7 µg/mL, SI = 5.5) and weak activity against A549 (IC_50_ = 157.1 ± 6.6 µg/mL, SI = 1.8). In contrast, all three EOs showed low selectivity and poor cytotoxicity in LoVo cells. Regarding combinations, REO + MEO and REO + SEO combinations significantly improved cytotoxicity against A549 cells with IC_50_ values of 11.11 ± 1.26 µg/mL (SI = 36.9) and 16.83 ± 4.32 µg/mL (SI = 16.5), respectively, compared to either EO alone. This enhancement was supported by FIC indices of 0.207 and 0.365, indicating a synergistic interaction in both cases.

These findings suggest that REO-based combinations may enhance the anticancer efficacy, particularly in lung cancer cells. Although the individual EOs exhibited weak activity against LoVo cells, all their combinations resulted in moderate cytotoxicity, with lower IC_50_ values and FIC indices indicating additive to synergistic effects. This highlights the complexity of molecular interactions between different EOs.

In the case of MCF7 cells, both the REO + MEO and REO + SEO combinations showed indifferent interactions (FIC indices of 3.940 and 2.414, respectively), despite their moderate cytotoxic activity. This suggests that the observed effects were primarily due to the action of individual oils, rather than a true combinatory enhancement. In contrast, the pairing of MEO and SEO was less effective against MCF7 cells (IC_50_ = 77.88 ± 4.15 µg/mL, SI = 2.9), exhibiting an antagonistic interaction (FIC index = 4.779), which indicates a reduction in activity when these oils were combined, despite their individual efficacy. Nevertheless, this same combination showed an additive effect on A549 (IC_50_ = 84.46 ± 13.45 µg/mL, SI = 2.6, FIC = 0.793), further emphasizing the cell target–specific nature of EO interactions.

### 2.3. In Vitro Antiviral Activity

The results of antiviral activity measurements of EOs tested individually and in a 1:1 combination against AdV5 and HSV-1 under suspension conditions according to the standard European procedure EN 14476, are presented in Table 3. Viruses were pretreated with EOs at 80 µg/mL (MNTC: maximum non-toxic concentration), which was determined by examining cellular morphological alterations in treated cultures compared with untreated control cultures, while their combined MNTCs were used for the combination test.

The virucidal tests showed that after a 60 min contact time, all tested EOs achieved a complete reduction in virus titers (≥4 log_10_). This indicates that our samples effectively inactivated all tested viruses. For AdV5, the highest virucidal effect was observed with REO alone, whereas for HSV-1, it was observed with REO alone and REO + SEO, with reductions > 4 log_10_ after both 15 and 60 min. Strong virucidal activity was also noted for AdV5 with individual MEO and SEO, as well as with REO + MEO and REO + SEO combinations, and for HSV-1 with individual MEO and SEO, REO + MEO, and MEO + SEO combinations, showing a 4 log_10_ inactivation within 15 min and >4 log_10_ within 60 min.

The MEO + SEO mixture achieved only a 1 log_10_ reduction in AdV5 titers at 15 min but exhibited a strong effect particularly after 60 min, achieving 4 log_10_ inactivation. Extending the contact time significantly enhanced the effects of MEO, REO + MEO, and MEO + SEO on both viruses, as well as REO + SEO on AdV5 and SEO on HSV-1. However, a reducing effect was observed for SEO against AdV5 with extended contact time. Although the FIC index was not calculated, the interactions between the EOs were assessed by comparing the antiviral effects of each EO alone and in combination.

When combined, REO + MEO and REO + SEO generally displayed indifferent interactions, with reductions similar to those of the most active single agents. Notably, REO + SEO showed a potential additive effect on HSV-1 at 15 min. In contrast, the MEO + SEO combination demonstrated a clear antagonistic interaction with AdV5 at 15 min, which was markedly lower than that of either oil alone. These results indicate that while most combinations do not enhance antiviral effects, certain pairings may interfere with efficacy or show additive potential, depending on the virus and exposure time.

### 2.4. In Silico Results

The binding energies of the selected terpenoids from REO, MEO, and SEO with the studied enzymes are shown in Table 4. Molecular docking (MD) studies revealed that all compounds exhibited favorable binding energies, ranging from −4.2 kcal/mol to −7.2 kcal/mol, indicating potential interactions with the studied targets. For cancer targets, isocaryophyllene showed the highest potential as a ligand in the binding domains of pyruvate dehydrogenase kinase 3 (PDK3) (PDB ID: 1Y8O) and signal transducer and activator of transcription 3 (STAT3) (PDB ID: 6NJS), with glide scores of −6.4 and −6.0 kcal/mol, respectively.

**Figure 5:**
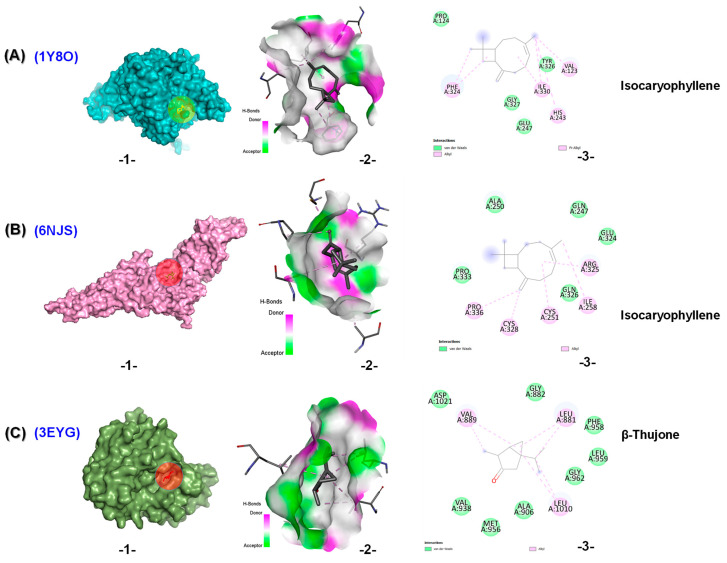
Molecular docking of (**A**) isocaryophyllene with PDK3 (PDB ID: 1Y8O), (**B**) isocaryopyllene with STAT3 (PDB ID: 6NJS), and (**C**) β-thujone with JAK1 (PDB ID: 3EYG). (**1**) Interaction of the molecule with the selected protein. (**2**) Surface view of the protein binding pocket occupied by the molecule. (**3**) 2D structural representation of protein residues interacting with the molecule.

In the active site of PDK3 (PDB ID: 1Y8O), isocaryophyllene formed four Van deer Waals interactions, as well as four hydrophobic interactions of the alkyl and pi-alkyl types (Figure 5A). Additionally, in the active site of STAT3 (PDB ID: 6NJS), it established five Van deer Waals interactions along with five alkyl liaisons (Figure 5B). β-Thujone demonstrated the highest potential in the binding domain of tyrosine-protein kinase (JAK1) (PDB ID: 3EYG), with a glide score of −6.3 kcal/mol. It stabilized by eight Van der Waals forces and three alkyl interactions as seen in Figure 5C.

**Figure 6:**
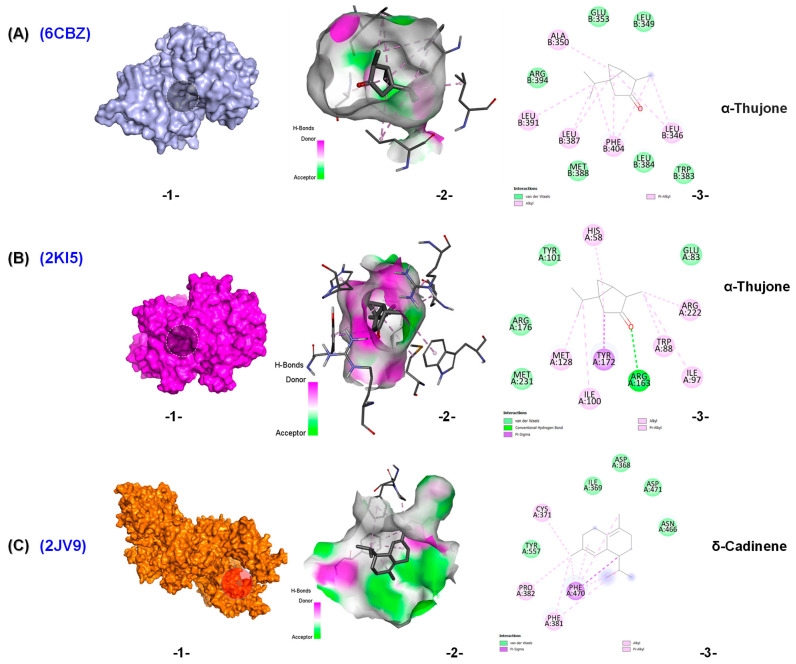
Molecular docking of (**A**) α-thujone with ERαLBD (PDB ID: 6CBZ), (**B**) α-thujone with HSV-1 thymidine kinase (PDB ID: 2KI5), and (**C**) δ-cadinene with HSV-1 DNA polymerase (PDB ID: 2JV9). (**1**) Interaction of the molecule with the selected protein. (**2**) Surface view of the protein binding pocket occupied by the molecule. (**3**) 2D structural representation of protein residues interacting with the molecule.

For viral targets, α-thujone exhibited strong binding potential as a ligand in the binding domains of HSV-1 thymidine kinase (PDB ID: 2KI5) and estrogen receptor alpha ligand binding domain (ERαLBD) (PDB ID: 6CBZ), with glide scores of −6.2 kcal/mol. The α-thujone-HSV-1 thymidine kinase was stabilized by four Van der Waals interactions, seven hydrophobic interactions (pi-sigma, alkyl, and pi-alkyl), and one hydrogen bond (Figure 6A). While the α-thujone-ERαLBD complex formed six Van der Waals interactions, five alkyl and pi-alkyl liaisons (Figure 6B). δ-Cadinene demonstrated the strongest binding potential in the domain of HSV-1 DNA polymerase (PDB ID: 2JV9), with a glide score of −7.2 kcal/mol.

Docking of δ-cadinene into the active site cavity of HSV-1 DNA polymerase revealed five Van der Waals interactions, as well as four hydrophobic bonds (pi-sigma, alkyl, and pi-alkyl) (Figure 6C). Overall, these MD results suggest that the selected terpenoids, especially isocaryophyllene, δ-cadinene, and thujones, possess promising binding affinities for viral and cancer-related proteins. Their interaction profiles support their potential role in modulating enzyme activity.

The predicted ADMET (Absorption, distribution, metabolism, excretion, and toxicity) properties of the tested molecules are shown in Table 5. These results demonstrated that the chosen compounds showed promising physicochemical properties and lipophilicity. All compounds displayed negative Log Kp (Logarithm of skin permeability coefficient) values, indicating limited skin permeability. γ-Terpineol, dihydro-, isomenthone, β-thujone, eucalyptol, α-thujone, camphor, menthone, and menthyl acetate showed high gastrointestinal (GI) absorption, whereas others had low absorption, potentially limiting their oral bioavailability. Most compounds were blood–brain barrier (BBB) permeants, except δ-cadinene, β-caryophyllene and isocaryophyllene, which could restrict their central nervous system activity. All selected compounds were P-glycoprotein (P-gp) non-substrates.

Regarding metabolic interactions with cytochrome P450 enzymes (CYP), none of the compounds inhibited CYP1A2 and CYP3A4. δ-Cadinene, β-caryophyllene and isocaryophyllene inhibited CYP2C19. α-Pinene, δ-cadinene, β-caryophyllene, β-pinene, camphene, isocaryophyllene, limonene, and menthyl acetate inhibited CYP2C9. These inhibitions were potentially leading to drug–drug interactions with drugs metabolized by these enzymes. *p*-Cymene was the only inhibitor of CYP2D6, suggesting a potential risk of drug–drug interactions involving this enzyme. All tested compounds had a bioavailability score of 0.55, suggesting moderate oral bioavailability.

In terms of elimination, the total clearance values ranged from 0.03 to 1.207, showing significant variations in elimination rates across the compounds. δ-Cadinene, β-caryophyllene, γ-terpineol, dihydro-, isocaryophyllene, eucalyptol, and menthyl acetate were cleared rapidly, while α-pinene, β-pinene, camphene, β-thujone, α-thujone, camphor, limonene, menthone, and *p*-cymene were eliminated much more slowly. Additionally, none of the compounds were substrates for organic cation transporter 2 (OCT2), indicating that they were not significantly excreted via this renal transporter.

**Figure 7:**
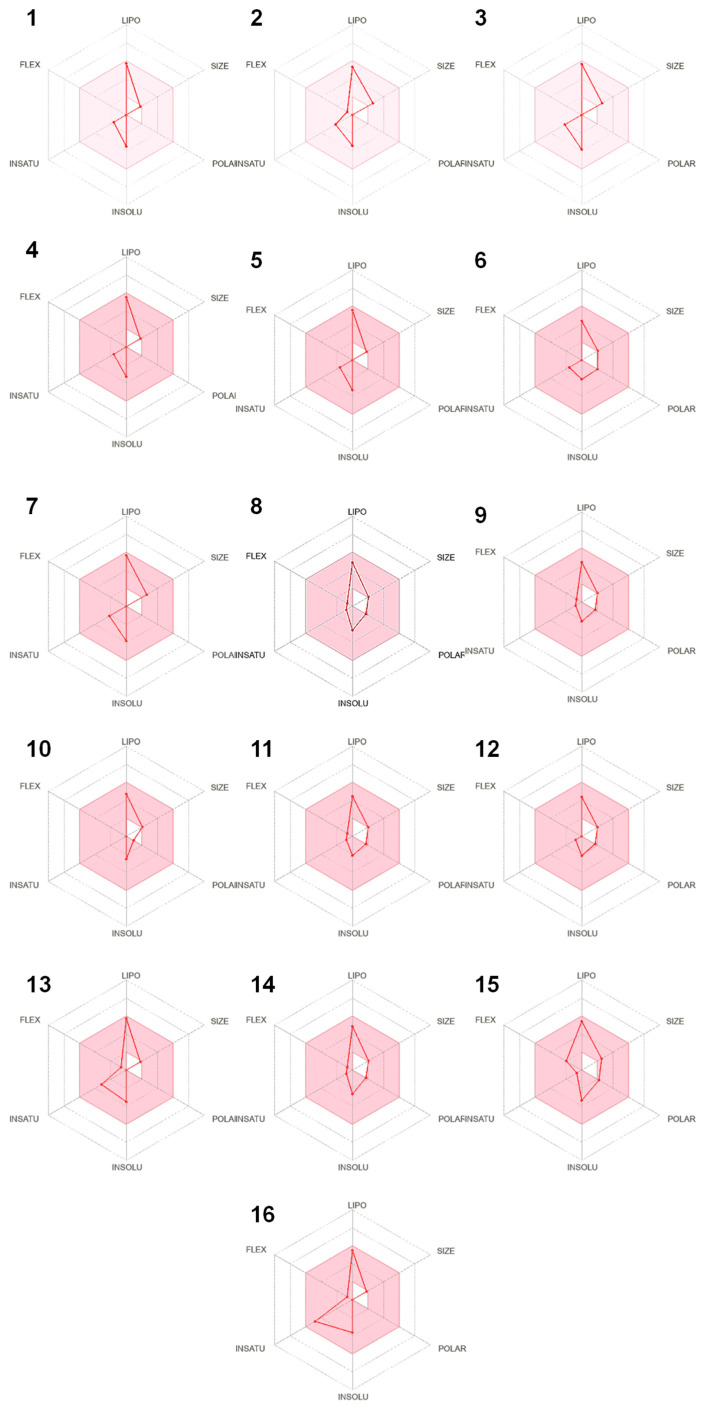
Bioavailability radar of the selected phytoconstituents. lipophilicity (Lipo), flexibility (Flex), insaturation (Insatu), size, polarity (Polar), and solubility (Insolu). (**1**): α-Pinene, (**2**): δ-Cadinene, (**3**): β-Caryophyllene, (**4**): β-Pinene, (**5**): Camphene, (**6**): γ-Terpineol, dihydro-, (**7**): Isocaryophyllene, (**8**): Isomenthone, (**9**): β-Thujone, (**10**): Eucalyptol, (**11**): α-Thujone, (**12**): Camphor, (**13**): Limonene, (**14**): Menthone, (**15**): Menthyl acetate, (**16**): *p*-Cymene.

**Figure 8:**
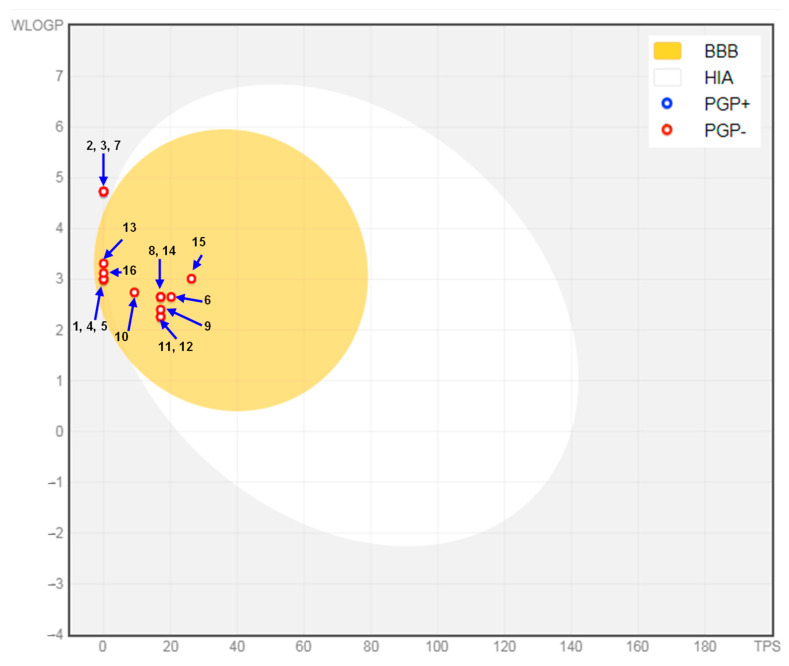
Boiled-egg graph of the selected phytoconstituents. 1: α-Pinene, 2: δ-Cadinene, 3: β-Caryophyllene, 4: β-Pinene, 5: Camphene, 6: γ-Terpineol, dihydro-, 7: Isocaryophyllene, 8: Isomenthone, 9: β-Thujone, 10: Eucalyptol, 11: α-Thujone, 12: Camphor, 13: Limonene, 14: Menthone, 15: Menthyl acetate, 16: *p*-Cymene.

The selected phytoconstituents met the ideal criteria (pink area in the bioavailability radar) for each of the six key characteristics (lipophilicity, flexibility, saturation, solubility, polarity, and size), suggesting a favorable prediction of oral bioavailability (Figure 7). Figure 8 presents the pharmacokinetic analysis, showing that all compounds exhibited a high probability of GI absorption and BBB permeability. However, δ-cadinene, β-caryophyllene, and isocaryophyllene were positioned outside both the yellow and white zones of the Boiled-Egg graph, indicating a low predicted absorption in the GI tract. In summary, γ-terpineol, dihydro-, isomenthone, β-thujone, eucalyptol, α-thujone, camphor, and menthone, exhibited the most favorable ADMET properties, aligning well with drug-like standards. In contrast, α-pinene, δ-cadinene, β-caryophyllene, β-pinene, camphene, isocaryophyllene, limonene, menthyl acetate, and *p*-cymene had limitations in their properties, which could reduce their therapeutic potential.

## 3. Discussion

In light of the ongoing global threat posed by pandemics, there is an urgent need to identify and evaluate alternative therapeutic agents, including natural compounds, such as EOs, for their cytotoxicity and antiviral activity against specific pathogens [14,15]. However, to ensure that the observed reduction in viral infectivity is directly attributed to the action of EOs on the virus, rather than its toxic effects on host cells, it is crucial to assess the cytotoxicity of the EO [16]. Accordingly, this study investigated the in vitro cytotoxicity and antiviral effects of EOs from three plants commonly grown and cultivated in Algeria, *R. officinalis*, *M. × piperita*, and *S. officinalis*, as well as their 1:1 combination. The EO composition profiles of these three Lamiaceae plants have been widely studied, revealing significant chemodiversity. It is well established that EO composition is significantly influenced by genetic, geographic, and environmental factors, leading to the identification of different chemotypes within the same plant species [17]. Consequently, we analyzed the composition of these oils from the Algerian specimens. In the literature, REO from various regions worldwide consistently identify eucalyptol, α-pinene, and camphor as major constituents, with concentration ranges of eucalyptol (20.88–51.79%), α-pinene (12.94–23.28%), and camphor (8.38–20%) [18,19,20,21,22]. In contrast, our REO exhibited high levels of camphor (23.52%) and α-pinene (21.20%) but comparatively lower amounts of eucalyptol (5.02%). For MEO, menthol is frequently reported as the dominant chemotype, with concentrations ranging from 19.55% to 43.25% [15,23,24]. However, our MEO contained γ-terpineol, dihydro- (43.50%) and menthone (25.97%) as the major compounds. Similarly, SEO is typically characterized by two main chemotypes, eucalyptol [21,22,25] and/or camphor [26,27], with α-thujone often appearing in significant amounts. Our SEO identified as a eucalyptol chemotype (23.57%) but also contained high levels of α-thujone (22.02%) and camphor (20.5%). It is worth noting that numerous studies have reported considerable variation in the concentrations of these compounds, underscoring the influence of different factors on EO composition. Our GC-MS analysis revealed that the three EOs shared twelve common components (α-thujene, α-pinene, sabinene, β-pinene, β-myrcene, α-terpinene, terpinolene, γ-terpinene, eucalyptol, terpinen-4-ol, α-terpineol, and caryophyllene oxide), although their relative percentages varied across the different EOs.

EOs have attracted growing interest as promising anticancer agents due to their diverse pharmacological effects [28]. As multi-target agents, EOs have significant potential as alternatives for chemoprevention and complementary cancer therapy by inducing apoptosis, inhibiting cell proliferation, and enhancing cancer cell sensitivity to chemotherapy [2,28]. In this investigation, to assess the concentration over which tested EOs would be cytotoxic for non-tumor cells, the NHDF cell line was used. All EOs were non-toxic to NHDF cells, indicating their safety in line with the European Medicines agency’s assessment [29,30,31]. The tested samples demonstrated high to moderate anticancer activity against MCF7 and A549, and low activity against LoVo cells. While individual EOs demonstrated strong anticancer effects against MCF7 cells, EO combinations were more effective against A549 and LoVo cells. This aligns with previous findings showing that MCF7 cells are generally more sensitive to EOs compared to other cancer cell lines [32]. The therapeutic potential for MCF7, A549, and LoVo cell lines on their ability to be absorbed, distributed, and reach the tumor site in sufficient concentrations within the human body [33]. In our study, REO (camphor chemotype) exhibited significant antiproliferative activity against MCF7 and A549 cell lines but showed low activity against LoVo cells. In the literature, the REO eucalyptol chemotype inhibited MCF7 cell proliferation with IC_50_ values of 0.253 µL/mL [34], 7.38 µg/mL [35], 190.1 µg/mL [36], and >100 µg/mL [37]. The REO squalene chemotype showed an IC_50_ value of 78 µL/mL in MCF7 cells [32]. Few studies have examined the effect of REO on A549 cells, but notable findings have included IC_50_ values of 3.06 µg/mL [35] and 8.50 µg/mL [38]. Furthermore, the REO eucalyptol chemotype from Palestine was reported to be non-toxic to multiple cell types [39]. Additionally, REO eucalyptol chemotype from Algeria showed lower anticancer activity against MCF7, A549, and colon carcinoma cell line (Caco-2) [40]. Wang et al. found that REO exhibited stronger activity than its individual major components, such as α-pinene, eucalyptol, and β-pinene [41]. Singh et al. and Taibi et al. confirmed that camphor exhibits significant anticancer activity against various cell lines [42,43]. This suggests that REO effectiveness likely results from the combined effects of both the major and minor compounds. MEO demonstrated low anticancer activity against A549 and LoVo cells and moderate activity against MCF7 cells. Thus, moderate to low anticancer activity has been reported for MEO against various cancer cell lines [7,37,44,45,46,47,48,49]. However, Sun et al. reported that the MEO menthol chemotype from China demonstrated significant anticancer effects against human gastric cancer (SGC-7901), human lung carcinoma (SPC-A1), and human leukemia (K562), with IC_50_ values of 38.79, 10.89, 16.16 µg/mL, respectively [50]. γ-Terpinene, a major compound in MEO, has been reported to exhibit low anticancer activity against various cell lines [51,52,53]. Similarly, menthone exhibited moderate anticancer activity, with an IC_50_ value of 80 µg/mL against MCF7 cells [54]. These findings suggest that the low-to-moderate antiproliferative effects of MEO may be attributed to these two major compounds. The studied SEO (eucalyptol chemotype) exhibited the strongest antiproliferative effect against MCF7 cells, moderate activity against A549 cells, and low activity against LoVo cells. This finding aligns with that of Kallel et al., where the SEO camphene chemotype from Tunisia inhibited MCF7 cell proliferation with an IC_50_ value of 3.12 µg/mL [55]. In contrast, the SEO eucalyptol chemotype demonstrated moderate anticancer activity against MCF7 cells, with an IC_50_ of >180 µg/mL [56]. The SEO camphor chemotype from Morocco showed lower anticancer activity against MCF7 cells, with an IC_50_ value of 554.4 µg/mL [57]. Similarly, the camphor chemotype of SEO from Algeria exhibited reduced anticancer activity against MCF7, A549, and Caco-2 cell lines [40]. Furthermore, the SEO *cis*-thujone chemotype inhibited MCF7 cell proliferation with an IC_50_ of >100 µg/mL [37]. Additionally, the eucalyptol chemotype of SEO from Lebanon was reported to be non-toxic to MCF7, human amelanotic melanoma (C32), and hormone-dependent prostate carcinoma (LNCaP) cell lines, and showed low toxicity against renal cell adenocarcinoma (ACHN) [58]. Wu et al. reported that eucalyptol and camphor, the major compounds of our SEO, showed low activity against MCF7 and A549 cells [53]. Overall, few studies have explored the effects of REO, MEO, and SEO on LoVo cells.

In summary, our results reveal that REO and SEO are the most effective and selective when used alone, particularly against breast cancer cells. Furthermore, their combinations, especially REO + MEO and REO + SEO, exhibit synergistic effects against A549 and LoVo cells, while maintaining low toxicity toward normal cells. These findings highlight the potential of EO combinations as promising candidates for the development of safer and more effective anticancer therapies. Interestingly, all combinations demonstrated lower cytotoxicity toward NHDF cells compared to individual EOs, suggesting that the combination strategy may reduce toxicity toward normal cells. This observation suggests that combining EOs can enhance anticancer efficacy, particularly against lung and colorectal cancer cells, while minimizing cytotoxicity to normal cells.

The virucidal effect of the tested EOs was assessed using the virion pretreatment method, which evaluates their ability to neutralize or inactivate the virus [59]. Antiviral activity results demonstrated that both individual EOs and their combinations met the standard disinfectant requirements for all tested viruses. All formulations significantly reduced the titers of AdV5 and HSV-1 after 1 h of incubation, achieving reductions of approximately 4 log_10_ and >4 log_10_. The most significant effects were observed with REO alone and the REO + SEO mixture against HSV-1, as well as REO alone against AdV5. Several studies support these findings, showing that the strongest antiviral effect occurs when viruses are incubated with substances for at least 1 h prior to their introduction to host cells, this indicates a direct virucidal action on cell free viruses [59]. The tested EOs effectively inhibited AdV5 and HSV-1 after both 15 and 60 min of exposure, suggesting that EOs directly inactivate the viruses by interacting with the surface proteins of AdV5 and the envelope proteins of HSV-1. This interaction likely impairs the viruses’ ability to attach to and enter host cells. Given that EOs can inhibit acyclovir-resistant HSV-1 strains, as shown in a study by Schnitzler et al., their mechanism of action differs from that of acyclovir, which targets the viral enzyme DNA polymerase [60]. Therefore, EOs operate through a distinct mechanism compared to synthetic antivirals (e.g., penciclovir, acyclovir), which focus specifically on the intracellular phase of viral replication. However, the exact mechanism of action of EOs remains unclear [59].

Our findings revealed that REO (camphor chemotype) was the most effective sample in reducing the infectious titers of both AdV5 and HSV-1 after just 15 min of exposure. This aligns with previous studies, which demonstrated REO’s efficiency in inactivating HSV-1 [61,62,63]. Additional studies have emphasized REO’s broad antiviral potential against various enveloped and non-enveloped viruses. For instance, the α-pinene chemotype from Canada reduced HSV-1 titers by 2.69 log_10_ and the Murine Norovirus 1 (MNV-1) titer by 2.10 log_10_, though it showed minimal reduction in the hepatitis A virus (HAV) [20]. Similarly, the eucalyptol chemotype from Italy achieved a 3 log_10_ reduction in HAV titers [18] but had limited efficacy against bovine viral diarrhea virus (BVDV) (0.75 log_10_ reduction) [22] and no effect on Feline Calicivirus [21]. The Greek eucalyptol chemotype, however, demonstrated activity against AdV35 [19]. Our study further highlights its effectiveness against HSV-1 and, for the first time, its activity against AdV5, with the camphor chemotype showing the highest activity among previously tested chemotypes. Notably, camphor itself is a well-established compound, valued for its therapeutic applications in respiratory depression, heart failure, and poisoning from soporific and narcotic agents [64]. Brand et al. reported that camphor alone exhibited moderate activity against HSV-1 [65]. Furthermore, various natural monoterpenes and their derivatives, including camphor, borneol, and camphene, have been recognized for their antiviral properties and contribution to antiviral drug development [64,66,67]. Supporting this, our REO sample contained 21.20% α-pinene, which has been shown to reduce HSV-1 infectivity by 96% [68]. Our MEO (γ-terpineol, dihydro- chemotype) displayed significant antiviral activity against both AdV5 and HSV-1, particularly after 1 h of contact, indicating a time-dependent virucidal effect. However, the literature highlights the differences in MEO activity among chemotypes. For example, the menthol chemotype from Iran reduced HSV-1 and Poliovirus 1 (PV1) titers by 4.5 log_10_ and 3.75 log_10_, respectively [24], whereas the same chemotype from Czechia showed no effect against SARS-CoV-2 [15]. The MEO menthol chemotype from Germany reduced HSV-1 infectivity by 82% and HSV-2 infectivity by 92% [23]. Other studies have validated the antiviral efficacy of MEO against HSV-1 [61,62]. However, MEO showed no activity against SARS-CoV-2 [15]. These results support established antiviral effects of MEO against certain enveloped and non-enveloped viruses, further supported by our study’s results against AdV5 and HSV-1. The tested SEO (eucalyptol chemotype) also exhibited significant antiviral effects against both AdV5 and HSV-1, with a time-dependent virucidal effect. Literature reports show varied activity of SEO eucalyptol chemotypes. For example, the Italian eucalyptol chemotype was ineffective against Feline Calicivirus [21] but exhibited a 0.75 log_10_ reduction against BVDV [22]. The Lebanese eucalyptol chemotype showed weak activity against SARS-CoV and no effect against HSV-1 [25]. In contrast, the SEO camphor chemotype from Egypt was highly effective against avian influenza virus (H5N1) [27], while the Spanish camphor chemotype demonstrated strong activity against HSV-1, albeit not exceeding the effect of eucalyptol alone [26]. Interestingly, while the SEO eucalyptol chemotype exhibited moderate activity against influenza A virus (H1N1), the same study suggested that eucalyptol may not be the primary component responsible for the antiviral activity of EOs like EOs from *Thymus vulgaris* and *Eucalyptus globulus* [69]. Overall, the observed antiviral effects of our EOs are likely due to the synergistic interactions of their major and minor components.

The antiviral potential of EOs is due to their chemical composition, including the types and contents of monoterpenes and sesquiterpenes they contain [15]. The major compound class in our EOs was monoterpenes, which are known to enhance cytoplasmic membrane permeability and fluidity, disrupt the organization of membrane-embedded proteins [19]. Since the monoterpenes in EOs are extracted together from plant tissues as a mixture, and their combined effects can include both synergistic and additive actions, potentially boosting their biological potentials [15]. However, these interactions among monoterpenes may also lead to increased cytotoxicity to human cells, emphasizing the need for careful evaluation of EO formulations [70]. Considering the synergistic effects, the antiviral activity became more pronounced after 60 min of exposure across combined EOs. The combination MEO + SEO exhibited greater antiviral potential than each individual EO. Although REO was the most effective antiviral agent on its own, it significantly enhanced the activity of the two combinations: REO + MEO and REO + SEO. However, the combination MEO + SEO did not demonstrate a similar effect compared to the REO-based mixtures. This discrepancy might be attributed to the distinct chemical compositions of MEO and SEO. It suggests that the interaction of REO compounds with MEO compounds in the REO + MEO mixture, as well as with SEO compounds in the REO + SEO mixture, increased the intensity of shared compounds, potentially explaining the observed enhancement in antiviral activity.

Understanding protein-ligand interactions is a fundamental aspect of structure-based drug design [71,72]. The congruence between in vitro activity and in silico predictions strengthens our understanding of the potential mechanisms of action and the therapeutic relevance of these phytocompounds. In the context of cancer, most human malignancies are linked directly or indirectly to the activation of signaling pathways [73]. This has driven the development of potent kinase inhibitors, which represent a promising and dynamic area in cancer therapeutics [73]. MD results revealed that all major compounds tested exhibited strong binding affinities towards the selected anticancer and antiviral receptors. Compounds γ-terpineol, dihydro- (present in MEO), isomenthone (present in MEO), β-thujone (present in SEO), eucalyptol (present in REO, MEO, and SEO), α-thujone (present in SEO), menthone (present in MEO), and menthyl acetate (present in MEO) exhibited the most favorable ADMET properties, aligning well with drug-like standards. In contrast, the remaining compounds have limitations in their properties, which could reduce their therapeutic potential. Among the tested compounds, β-thujone emerged as the most effective cancer enzyme inhibitor, as evidenced by its minimum energy values and favorable drug-likeness and pharmacokinetic profile. Furthermore, in the context of viral diseases, in silico analyses suggested strong interactions between γ-terpineol, dihydro-, isomenthone, eucalyptol, β-thujone, and α-thujone and viral enzymes, indicating their potential to disrupt key pathways associated with viral progression. These findings highlight the potential of EOs from *S. rosmarinus*, *M. × piperita*, and *S. officinalis* as sustainable sources of novel therapeutic agents for anticancer and antiviral applications.

Comparing the results of this study with others is difficult because of the varying chemical profiling of EOs, which influences factors such as harvest period, extraction methods, drying method, and compound degradation. Additionally, plant characteristics, such as age, growth conditions and meteorological factors, can influence the consistency of this investigation with previous research. These variations hinder the standardization of EO protocols. Furthermore, the experiments evaluating the anticancer and antiviral activities of EOs involve different methodologies, adding another layer of complexity. Nevertheless, these findings highlight the potential of EO combinations to achieve enhanced efficacy while underscoring the importance of understanding the chemical interactions that drive their anticancer and antiviral activities. The precise mechanism underlying the observed effects of the tested oils and their combinations remain unclear, and further research is required to elucidate their mechanisms of action.

## 4. Materials and Methods

### 4.1. Botanical Material

Samples of the aerial parts of *S. rosmarinus* and *M. × piperita* (stems, leaves, and flowers) were collected from their natural habitats in eastern Algeria, while those of *S. officinalis* were harvested from a cultivated area during the flowering stage. Detailed information on their scientific and vernacular names, collection origins, geographic coordinates, voucher specimen numbers, and harvest periods is provided in Table 6. The plants were authenticated by Dr. Azzeddine Zeraib, Doctor of Botany at the Department of Agronomy, Khenchela University, Algeria. Voucher specimens were deposited at the Herbarium of Khenchela University, Algeria. Fresh samples were dried in shade at room temperature. EOs were extracted from crushed, dried samples by steam distillation over a 90 min extraction period, according to the protocol described by Petrović et al. [74]. The distillation temperature was set at 104 ± 5 °C. The extraction yield was recorded, and the obtained EOs were stored in amber vials at −18 °C for use in upcoming experiments.

### 4.2. Essential Oil Analysis

The volatile compounds in REO and MEO were analyzed using GC-MS on a Shimadzu GC-MS QP 2020 system (Kyoto, Japan). Separation was achieved on a Zebron ZB-5 MSi, capillary column (30 m × 0.25 mm × 0.25 µm; Phenomenex, Torrance, CA, USA). The column oven temperature was programmed to increase from 50 °C at a rate of 3 °C/min to a final temperature of 250 °C. Helium was used as the carrier gas at a flow of 0.93 mL/min, with a split of 50 and a linear velocity of 35 cm/s. 1 µL of EO/ultrapure cyclohexane (20 µL EO dissolved in one mL of cyclohexane) was injected at 250 °C. The total GC-MS program was 69.67 min. The volatile compounds in SEO were analyzed using a Bruker SCION 436-GC MS/MS (SCION Fermont, CA, USA) equipped with an SH-5 MSi capillary column (30 m × 0.25 mm × 0.25 µm; Shimadzu, Kyoto, Japan). Approximately 20 µL of EO was dissolved in one mL of cyclohexane (2% *v*/*v*) and injected at a volume of 1 µL at 220 °C. Helium served as the carrier gas at a flow of 1 mL/s and a split ratio of 50. The GC oven temperature program began at 50 °C, increased to 160 °C at a rate of 1 °C/min, then to 300 °C at a rate of 15 °C/min, and was held for 3 min. The total program was 127.99 min. MS analysis was conducted with scans ranging from 40 to 450 *m*/*z* at a scan rate of 3 scans/s, with both the ion source and interface temperatures maintained at 250 °C.

Volatile compounds in REO, MEO, and SEO were identified by comparing their calculated Kovats Retention Indices (KI) and linear retention indices with reference libraries: Flavors and Fragrances of Natural and Synthetic Compounds (FFNSC 3.0) and the National Institute of Standards and Technology (NIST 20). KI values were determined using a macro developed by Lucero et al. [75] using the retention times (RT) of C_8_–C_24_ n-alkanes (Sigma-Aldrich, Steinheim, Germany) as reference compounds, which were injected under the same chromatographic conditions. Compound quantification was achieved by the peak area normalization method, using the peak area of the individual compounds. A mass spectral similarity of ≥80% was used as the identification criterion. Data processing was carried out using the GC-MS Postrun Analysis software version 4.45 (Shimadzu, Kyoto, Japan) and AMDIS GC-MS Analysis version 2.73.

### 4.3. In Vitro Cytotoxic Activity

#### 4.3.1. Cell Lines

MCF7 (ECACC: 86012803-1VL), A549 (ECACC: 86012804-1VL), LoVo (ATCC: Manassas, VA, USA), and NHDF (Lonza: CC-2511) were used for cytotoxicity evaluation. The cells were maintained in corning culture flasks with the recommended medium under standard conditions at 37 °C in a humidified atmosphere with 5% CO_2_, in a CO_2_ incubator (Thermo Fisher Scientific, Waltham, MA, USA). They were examined at least twice a week under a microscope and subcultured using TrypLE solution, or the appropriate medium was changed. Cancer cell lines were grown in Eagle’s Minimum Essential Medium (EMEM) (Sartorius AG, Goettingen, Germany). NHDF cells were grown in Dulbecco’s modified Eagle medium and Ham’s Nutrient Mixture F-12 (DMEM/Hams F-12) (Capricorn Scientific GmbH, Ebsdorfergrund, Germany). All media were supplemented with 10% fetal bovine serum (FBS), 50 mg/mL Gentamicin Sulfate (Sartorius AG, Goettingen, Germany), 200 mM Ultraglutamine 1 (Lonza, Verviers, Belgium), and 250 µg/mL Amphotericin B (Fungizone, Gibco Life Technologies, Grand Island, NY, USA). The collected cells used in this assay were obtained from the European Collection of Authenticated Cell Cultures (ECACC, UK Health Security Agency, Porton Down, Salisbury, UK) and the American Type Culture Collection (ATCC, Rockville, MD, USA).

#### 4.3.2. Cell Culture Preparation

Cells in the logarithmic growth phase (at 90% confluence) were utilized for this procedure. They were detached by treating them with TrypLE solution (gibco Life Technologies, USA) for 10 min at 37 °C. An equal volume of the appropriate medium for the cell line was added to deactivate the TrypLE solution and neutralize the trypsin’s effect. The cell suspension was then transferred into a tube and centrifuged for 5 min at 400 rpm (Thermo Fisher Scientific, Waltham, MA, USA). After centrifugation, the supernatant was discarded, and fresh medium was added to the cell pellet. Cell counting was performed according to the trypan blue exclusion method (T8154, Sigma Aldrich, UK) [76], using the JuLI Br Live Cell Movie Analyzer (NanoEntek, Seoul, Republic of Korea) and Countess Cell Count Chamber Slides (Invitrogen, Thermo Fisher Scientific, Waltham, MA, USA). The cells were subsequently seeded into 96-well plates (Eppendorf Cell Culture Plate, Eppendorf AG, Hamburg, Germany) at a density of 2 × 10^4^ cells/well in 200 µL of medium and maintained to regenerate at 37 °C for 24 h in an incubator supplied with 95% humidity and 5% CO_2_, allowing them to attach to the surface before treatment with EOs.

#### 4.3.3. Cytotoxicity Assay

The cytotoxicity evaluation of individual and combined EOs was determined using the Sulforhodamine B (SRB) method, according to The US National Cancer Institute 60 human tumor cell line anticancer drug screen (NCI60) [77], with slight modifications. A 32 mg/mL stock solution of the tested EOs in DMSO was initially prepared. Just before their addition to cell cultures, this solution was diluted with phosphate-buffered saline (PBS) (Lonza, Walkersville, MD, USA) to a concentration of 3.2 mg/mL. For the combined EOs, various associations were prepared by mixing equal volumes of any two EOs. These mixtures were then used in the cytotoxicity bioassay to assess the potential synergistic, additive, or antagonistic interactions between the EOs. Cells were incubated for 48 h with increasing concentrations of EOs. Sample volumes added to each microtiter well were 20, 10, 5, 2, and 1 µL to obtain final concentrations of 320, 160, 80, 30 to 15 µg/mL, respectively. In this context, the DMSO concentration did not exceed 1% and did not affect cell viability compared to the untreated control, confirming its suitability as a vehicle control in preliminary experiments. Cells treated with maintenance medium alone served as a negative control. Five wells were used for each dilution and each control assay.

During the incubation period, cells were observed using a ZEISS Primovert inverted cell culture microscope (Carl Zeiss Suzhou Co., Ltd., Suzhou, China). After incubation, the contents of the wells were fixed by adding 35 µL of 50% (*w*/*v*) cold trichloroacetic acid solution (TCA) and incubated for 20 min at 4 °C. The supernatant was then removed, and the plates were washed five times with tap water and dried at room temperature. To each well, 50 µL of 0.4% (*w*/*v*) SRB solution was added and incubated for another 20 min at room temperature to stain cell proteins. After staining, the unbound dye was rinsed five times with 1% acetic acid solution, and the plates were air-dried again for two days. The protein-bound dye was dissolved in 100 µL of 10 mM Trizma base for 15 min, and the absorbance was measured at 515 nm using a MultiscanGo reader (Thermo Scientific, Waltham, MA, USA).

The cell growth percentage (CG%) was calculated at each concentration of the test EOs using absorbance measurements taken at time zero (Tz), control growth (*C*), and growth in the presence of test EOs at various concentrations (*Ti*) following Equation (1) [77].

(1) $$\mathrm{CG}\%=\frac{\left(Ti-Tz\right)}{\left(C-Tz\right)}\times 100$$

According to the NCI60 [77], 100% represents control growth, 0% indicates complete inhibition of growth (cytostasis), and −100% corresponds to complete cell kill. Percent control growth values below 100% indicate growth inhibition, whereas values below 0% reflect net cell death. Conversely, values above 100% represent hormetic stimulation of cell growth. The cell growth inhibition concentration, which represents the concentration of EO required to reduce cell growth by 50% (GI_50_ or IC_50_), was derived from the graphs. The MNTC was used for further testing. The SI was determined using Equation (2). Where an SI ≥ 2.0 is deemed to be indicative of promising selectivity, as proposed by Suffness et al. [78].

(2) $$\mathrm{SI}=\frac{{IC}_{50}^{normal\;cell\;\left(NHDF\right)}}{{IC}_{50}^{tumour\;cell\;\left(Cancer\;cell\right)}}\;$$

### 4.4. In Vitro Antiviral Assay

#### 4.4.1. Viruses

Virucidal properties were determined against enveloped virus (HSV-1, ATCC VR-1779) and non-enveloped virus (AdV5, ATCC VR-1516). For AdV5 amplification, A549 (ATCC CRM-CCL-185) cultured in EMEM (Lonza, Basel, Switzerland) were used. For HSV-1 amplification, human cervix carcinoma cells (Hela, ATCC CRM-CCL-2) were propagated in DMEM (Capricorn Scientific GmbH, Ebsdorfergrund, Germany). The media were enriched with 10% of FBS, 4 mM of L-glutamine (Biological Industries, Kibbutz Beit-Haemek, Israel), 100 U/mL of penicillin, and 100 g/mL of streptomycin (Sigma-Aldrich, Munich, Germany). The isolated strains of the test viruses and cells used in this assay were obtained from the American Type Culture Collection-ATCC (Rockville, MD, USA).

#### 4.4.2. Virus Inoculum

On the day of infection, after removing the growth medium from the cell culture, cell monolayers at 80% confluence were infected with viruses at a multiplicity of infection (TCID_50_/_mL_ = 10^−6^) and incubated for 2 h at room temperature. After washing with Dulbecco’s phosphate-buffered saline (DPBS) (Capricorn Scientific GmbH, Ebsdorfergrund, Germany) to remove unadsorbed virus, the infected cells were resuspended in fresh growth medium enriched with 1% FBS and incubated for 3–6 days at 37 °C and 5% CO_2_ until viral cytopathic effect (CPE) was observed. The collected medium was centrifuged for 15 min at 3000 rpm and transferred to fresh flasks to obtain a clear virus suspension.

#### 4.4.3. Antiviral Assay

The antiviral activity was evaluated according to European Standard EN 14476 [79]. This standard outlines a quantitative suspension test used to assess virucidal effectiveness within the medical field by mixing the virus suspension and interfering substances with the investigated substance at a ratio of 1:1:8. Samples were collected at designated contact times, and residual infectivity was defined.

In this investigation, the tested samples and the virus suspension were incubated with the interfering substance, PBS, at room temperature for 60′ and 15′ min. The obtained mixtures were then serially diluted tenfold up to 10^−11^ in the medium. 50 µL of each dilution, in three replicates, was transferred to a microtiter plate (Thermo Scientific, Denmark) containing freshly trypsinized A549 or HeLa cells (10^−15^ × 10^3^ cells per well). This produced final concentrations ranging between 30 µg/mL and 3 × 10^−9^ µg/mL, from the first to the 12th well. In addition, cells treated with no agent served as a negative control (cell control CC), whereas serial tenfold dilutions of the virus suspension in culture mediums, ranging from 1 to 10^−11^, served as a positive control (virus control VC). Immediately at the end of the contact period, the activity of the disinfectant was stopped by dilution to 10^−11^. Plates were incubated at 37 °C with 5% CO_2_, and observed every day for up to 6 days until the viral CPE was detected in the VC wells, using an inverted microscope (Olympus Corp., Hamburg, Germany; Axio Observer, Carl Zeiss Microscopy Deutschland GmbH, Jena, Germany).

As stated in EN 14476, a substance is deemed to have virucidal effectiveness if, within the designated exposure period, the viral titter is reduced by ≥4 log10 steps (inactivation ≥ 99.99%). The infective dose (TCID_50_/_mL_) was calculated using the Spearman-Karber method [80] and the following formula (3):

(3) $$-\;{log}_{10\;}{TCID}_{50}=x^{0}-0.5+\;\sum \frac{r}{n}$$

where $x^{0}$ is the log_10_ of the lowest dilution with 100% positive reaction, *r* is the number of positive determinations of the lowest dilution step with 100% positive and all higher positive dilution steps, and *n* the number of determinations for each dilution step.

### 4.5. Determination of Interaction Effects for Essential Oil Combinations

The interaction effect between binary combinations of EOs in the cytotoxicity bioassay was evaluated using the FIC index, as described in Equation (4). Based on the FIC value, the interaction was classified as synergistic (FIC ≤ 0.5), additive (FIC > 0.5 to ≤1), indifferent (FIC > 1 to ≤4), or antagonistic (FIC > 4) [81].

(4) $$\mathrm{FIC}\;\mathrm{Index}={FIC}_{A}\;{+FIC}_{B}$$

where FIC_A_ = $\frac{{IC}_{50}\;of\;A\;in\;combination\;/\;2}{{IC}_{50}\;of\;A\;alone}$, FIC_B_ = $\frac{{IC}_{50}\;of\;B\;in\;combination\;/\;2}{{IC}_{50}\;of\;B\;alone}$.

A and B represent the tested EOs. As the EOs were combined in equal proportions (50% each), the IC_50_ values in the combination were halved for the FIC calculation. FIC analysis was not applicable in the antiviral bioassay due to the absence of dose–response data.

### 4.6. In Silico Analysis

The potential anticancer and antiviral properties of the major compounds in our EOs were explored in silico through MD analyses against six different target proteins using Autodock 4.2.6 software with the Lamarckian genetic algorithm (LGA) running for 10 iterations. The 3D structures of the major compounds were downloaded from the PubChem database (https://pubchem.ncbi.nlm.nih.gov/, accessed on 30 November 2024) and processed with ChemDraw software v14. The ligands underwent preparation, which included energy minimization, the addition of hydrogen atoms, and the application of necessary charges. The optimized 3D structures of these compounds were saved in PDB file format using OPENBABEL software, ensuring they were ready for docking analysis. The Protein Data Bank (PDB) (https://www.rcsb.org, accessed on 30 November 2024) was used to obtain high-resolution crystal structures. The target proteins for anticancer potential include PDK3 (PDB ID: 1Y8O), STAT3 (PDB ID: 6NJS), and JAK1 (PDB ID: 3EYG). For antiviral potential, the target proteins included ERαLBD (PDB ID: 6CBZ), HSV-1 thymidine kinase (PDB ID: 2KI5), and HSV-1 DNA polymerase (PDB ID: 2JV9). These proteins were refined and energy-optimized prior to docking. The active regions of these proteins where amino acids interact with the ligands were identified and a grid box was created to cover these regions. Finally, docking scores were reported in kcal/mol, docking analysis and 3D visualizations were conducted using Biovia Discovery Studio Visualizer to gain deeper insights into the ligand-protein interactions.

The selected constituents were analyzed for ADMET profile and drug-likeness prediction using the web-based server SwissADME (http://www.swissadme.ch/, accessed on 20 December 2024) and admetSAR (https://lmmd.ecust.edu.cn/admetsar2, accessed on 20 December 2024). The analysis included physicochemical properties (topological polar surface area, (TPSA)), pharmacokinetics (absorption: GI absorption and skin permeability; distribution: BBB permeant, and P-gp substrate; metabolism with CYP enzymes: CYP1A2 inhibitor, CYP2C19 inhibitor, CYP2C9 inhibitor, CYP2D6 inhibitor, and CYP3A4 inhibitor; excretion: total clearance and renal OCT2 substrate; toxicity: AMES (Bacterial reverse mutation assay) toxicity and hepatotoxicity), drug-likeness (bioavailability score), and lipophilicity (consensus Log P). Drug-likeness was verified using Lipinski’s rule [82] and Veber’s rule [83].

### 4.7. Statistical Analysis

Data are presented as mean and standard deviation. All graphs were prepared in Excel. The statistical evaluation was conducted using Statistica v13.3 software. Data were normally distributed and had equal variance, allowing for the use of one-way ANOVA with Tukey’s post hoc test (HSD). Significance was set at *p* < 0.05.

## 5. Conclusions

The rising burden of cancer and viral infections has underscored the urgent need for novel, safe, and effective therapeutic agents. This study highlights the promising anticancer and antiviral potential of EOs from *S. rosmarinus*, *M. × piperita*, and *S. officinalis*, along with their combinations. The consistent activity observed across in vitro and in silico platforms underscores their therapeutic potential and supports further investigation in drug development. Notably, REO, alone or in combination, exhibited broad-spectrum biological activity, positioning it as a particularly promising candidate for anticancer and antiviral applications. However, the underlying mechanisms of action of these EOs and their major constituents remain insufficiently validated in vivo, warranting further comprehensive studies. This research advocates for the strategic combination of EOs as a natural, non-chemical approach to enhance their efficacy and physicochemical properties.

## Figures and Tables

**Table 1:** GC-MS analysis of *S. rosmarinus, M. × piperita*, and *S. officinalis* essential oils.

N°	Compounds	Identif	Cf	KI Lit	REO			MEO			**SEO**		
					%	KI Exp	RT (min)	%	KI Exp	RT (min)	**%**	**KI Exp**	**RT (min)**
1	*trans*-2-Hexenal	MS, KI, S	Ad	854	-	-	-	-	-	-	0.12		6.25
2	1-Hexanol	MS, KI, S	Al	868	-	-	-	0.107	872	5.808	-	-	-
3	Furan, 2,5-diethyltetrahydro-	MS, KI	Td	897	-	-	-	0.047	898	6.608	-	-	-
4	Bornylene	MS, KI	Hm	908	0.017	907	6.883	-	-	-	-	-	-
5	Tricyclene	MS, KI	Hm	925	1.01	926	7.45	-	-	-	0.08	922	9.319
6	α-Thujene	MS, KI, S	Hm	929	0.081	932	7.617	0.037	931	7.608	0.31	929	9.655
7	α-Pinene	MS, KI, S	Hm	937	**21.202**	939	7.858	1.22	938	7.842	**2.13**	935	10
8	Camphene	MS, KI, S	Hm	952	**18.943**	954	8.375	-	-	-	**4.17**	949	10.853
9	Dehydrosabinene	MS, KI	Hm	956	0.014	959	8.567	-	-	-	-	-	-
10	Sabinene	MS, KI, S	Hm	974	0.012	977	9.283	0.268	977	9.275	0.33	974	12.561
11	β-Pinene	MS, KI, S	Hm	979	**3.275**	980	9.4	0.844	980	9.392	**3.1**	976	12.704
12	Vinyl amyl carbinol	MS, KI, S	Al	980	-	-	-	-	-	-	0.28	982	13.158
13	β-Myrcene	MS, KI, S	Hm	991	0.839	993	9.958	0.046	993	9.958	**2.2**	992	14.001
14	3-Octanol	MS, KI, S	Al	994	-	-	-	0.089	997	10.125	-	-	-
15	α-Phellandrene	MS, KI, S	Hm	1005	0.357	1006	10.483	-	-	-	-	-	-
16	3-Carene	MS, KI, S	Hm	1011	0.042	1012	10.717	-	-	-	-	-	-
17	α-Terpinene	MS, KI, S	Hm	1017	0.544	1019	10.975	0.044	1019	10.967	0.22	1016	15.869
18	*o*-Cymene	MS, KI, S	Hm	1022	-	-	-	0.169	1028	11.292	-	-	-
19	*p*-Cymene	MS, KI	Hm	1025	**2.661**	1028	11.3	-	-	-	0.59	1024	16.541
20	Limonene	MS, KI, S	Hm	1030	**3.265**	1032	11.475	1.456	1032	11.467	-	-	-
21	Eucalyptol	MS, KI, S	Om	1032	**5.023**	1034	11.558	**4.985**	1034	11.55	**23.57**	1030	17.034
22	*cis*-β-Ocimene	MS, KI, S	Hm	1038	0.051	1042	11.875	-	-	-	-	-	-
23	*trans*-β-Ocimene	MS, KI, S	Hm	1049	0.023	1053	12.317	0.027	1042	11.867	-	-	-
24	γ-Terpinene	MS, KI, S	Hm	1060	0.594	1063	12.758	0.083	1063	12.758	0.48	1060	19.691
25	*trans*-Sabinene hydrate	MS, KI, S	Om	1070	-	-	-	0.114	1070	13.1	0.18	1067	20.432
26	Terpinolene	MS, KI, S	Hm	1088	0.423	1090	14.05	0.071	1090	14.042	0.37	1087	22.549
27	*cis*-Sabinene hydrate	MS, KI, S	Om	1093	-	-	-	-	-	-	0.11	1095	23.518
28	Linalool	MS, KI, S	Om	1099	0.084	1101	14.575	0.092	1101	14.567	-	-	-
29	α-Thujone	MS, KI, S	Om	1103	-	-	-	-	-	-	**22.02**	1102	24.287
30	Fenchol	MS, KI, S	Om	1113	0.14	1115	15.15	-	-	-	-	-	-
31	β-Thujone	MS, KI, S	Om	1114	-	-	-	-	-	-	**4.37**	1113	25.389
32	*cis*-2-*p*-Menthen-1-ol	MS, KI	Om	1122	-	-	-	0.025	1124	15.508	-	-	-
33	α-Campholenal	MS, KI	Om	1125	0.044	1129	15.733	-	-	-	-	-	-
34	*trans*-Pinocarveol	MS, KI	Om	1139	0.042	1142	16.3	-	-	-	0.12	1136	27.674
35	Camphor	MS, KI	Om	1142	**23.517**	1148	16.55	-	-	-	**20.5**	1140	28.189
36	*cis*-Sabinol	MS, KI, S	Om	1143	-	-	-	0.033	1143	16.325	-	-	-
37	Isopulegol	MS, KI, S	Om	1146	-	-	-	0.134	1149	16.575	-	-	-
38	Camphene hydrate	MS, KI, S	Om	1148	0.068	1152	16.708	-	-	-	-	-	-
39	Menthone	MS, KI	Om	1154	-	-	-	**25.968**	1158	16.992	-	-	-
40	5-Methylundecane	MS, KI	Hy	1156	-	-	-	0.085	1163	17.225	-	-	-
41	Pinocarvone	MS, KI, S	Om	1164	0.028	1166	17.375	-	-	-	-	-	-
42	Isomenthone	MS, KI, S	Om	1164	-	-	-	**9.613**	1168	17.45	-	-	-
43	*trans*-Pinocamphone	MS, KI	Om	1160	-	-	-	-	-	-	0.14	1157	30.049
44	*endo*-Borneol	MS, KI	Om	1167	1.574	1169	17.508	-	-	-	0.8	1162	30.721
45	δ-Terpineol	MS, KI	Om	1166	-	-	-	-	-	-	0.58	1165	31.05
46	Isopinocamphone	MS, KI	Om	1173	-	-	-	-	-	-	0.08	1169	31.563
47	Menthol	MS, KI, S	Om	1174	-	-	-	0.283	1176	17.858	-	-	-
48	γ-Terpineol, dihydro-	MS, KI, S	Om	1178	-	-	-	**43.502**	1177	17.908	-	-	-
49	Terpinen-4-ol	MS, KI, S	Om	1182	0.967	1180	18.05	0.212	1180	18.058	0.5	1174	32.157
50	*p*-Cymen-8-ol	MS, KI, S	Om	1183	0.028	1188	18.433	-	-	-	0.08	1184	33.35
51	Isoneomenthol	MS, KI	Om	1188	-	-	-	0.673	1191	18.583	-	-	-
52	α-Terpineol	MS, KI, S	Om	1189	0.645	1192	18.667	0.063	1192	18.675	0.5	1187	33.84
53	Myrtenal	MS, KI, S	Om	1193	0.031	1197	18.925	-	-	-	0.11	1190	34.21
54	Pulegone	MS, KI, S	Om	1237	-	-	-	1.035	1242	20.842	-	-	-
55	Cuminaldehyde	MS, KI, S	Ad	1239	-	-	-	-	-	-	0.14	1236	39.527
56	Carvone	MS, KI, S	Om	1246	-	-	-	0.052	1247	21.067	-	-	-
57	Piperitone	MS, KI, S	Om	1253	-	-	-	0.166	1258	21.533	-	-	-
58	Neomenthyl acetate	MS, KI	Om	1274	-	-	-	0.145	1278	22.458	-	-	-
59	Bornyl acetate	MS, KI	Om	1285	0.314	1288	22.933	-	-	-	0.15	1282	45.302
60	Dihydroedulan	MS, KI, S	Bz	1293	-	-	-	0.029	1290	23.033	-	-	-
61	Menthyl acetate	MS, KI, S	Om	1295	-	-	-	**5.981**	1295	23.292	-	-	-
62	Carvacrol	MS, KI, S	Om	1299	0.018	1305	23.733	-	-	-	-	-	-
63	Isomenthol acetate	MS, KI	Om	1305	-	-	-	0.065	1309	23.925	-	-	-
64	α-Cubebene	MS, KI	Hs	1351	0.034	1353	25.742	-	-	-	-	-	-
65	Ylangene	MS, KI	Hs	1372	0.24	1374	26.683	0.021	1374	26.667	-	-	-
66	α-Copaene	MS, KI	Hs	1376	1.061	1379	26.875	0.026	1379	26.875	-	-	-
67	β-Bourbonene	MS, KI	Hs	1384	0.055	1387	27.258	0.056	1387	27.258	-	-	-
68	β-Elemene	MS, KI	Hs	1391	-	-	-	0.031	1394	27.575	-	-	-
69	Isocaryophyllene	MS, KI	Hs	1406	-	-	-	-	-	-	**3.21**	1406	60.811
70	β-Caryophyllene	MS, KI, S	Hs	1419	**4.169**	1422	28.725	0.726	1422	28.717	-	-	-
71	β-Copaene	MS, KI	Hs	1432	0.098	1432	29.125	-	-	-	-	-	-
72	α-Caryophyllene	MS, KI	Hs	1454	-	-	-	0.02	1457	30.133	1.73	1452	64.835
73	*trans*-α-Bergamotene	MS, KI	Hs	1435	0.029	1439	29.392	-	-	-	-	-	-
74	Aromadendrene	MS, KI	Hs	1440	0.051	1442	29.533	-	-	-	0.1	1449	65.683
75	*trans*-β-Farnesene	MS, KI	Hs	1457	0.131	1460	30.267	0.054	1460	30.275	-	-	-
76	*cis*-Muurola-4(14),5-diene	MS, KI	Hs	1463	0.035	1465	30.458	-	-	-	-	-	-
77	Cadina-1(6),4-diene	MS, KI, S	Hs	1481	0.038	1477	30.967	-	-	-	-	-	-
78	γ-Muurolene	MS, KI	Hs	1477	1.207	1480	31.092	-	-	-	0.15	1469	68.023
79	Germacrene D	MS, KI, S	Hs	1481	-	-	-	0.224	1484	31.267	-	-	-
80	α-Amorphene	MS, KI	Hs	1482	0.048	1483	31.225	-	-	-	-	-	-
81	α-Curcumene	MS, KI	Hs	1483	0.072	1486	31.342	-	-	-	-	-	-
82	β-Selinene	MS, KI	Hs	1486	0.108	1489	31.483	-	-	-	-	-	-
83	Bicyclogermacrene	MS, KI	Hs	1495	-	-	-	0.105	1499	31.933	-	-	-
84	γ-Amorphene	MS, KI	Hs	1496	0.374	1497	31.842	-	-	-	-	-	-
85	α-Muurolene	MS, KI	Hs	1499	0.502	1502	32.058	-	-	-	-	-	-
86	β-Alaskene	MS, KI	Hs	1499	-	-	-	-	-	-	0.29	1502	72.169
87	β-Bisabolene	MS, KI, S	Hs	1509	0.369	1511	32.383	-	-	-	-	-	-
88	γ-Cadinene	MS, KI	Hs	1513	0.923	1517	32.608	-	-	-	-	-	-
89	Calamenene	MS, KI	Hs	1523	-	-	-	-	-	-	0.12	1512	73.184
90	β-Cadinene	MS, KI	Hs	1518	-	-	-	-	-	-	0.13	1516	73.608
91	δ-Cadinene	MS, KI	Hs	1524	**2.38**	1527	32.975	0.048	1526	32.967	-	-	-
92	Cubenene	MS, KI	Hs	1532	0.129	1536	33.325	-	-	-	-	-	-
93	α-Cadinene	MS, KI	Hs	1538	0.124	1541	33.542	-	-	-	-	-	-
94	α-Calacorene	MS, KI	Hs	1542	0.211	1547	33.75	-	-	-	-	-	-
95	Caryophyllene oxide	MS, KI, S	Os	1581	0.334	1586	35.317	0.081	1586	35.317	0.45	1569	79.588
96	Viridiflorol	MS, KI, S	Os	1591	-	-	-	0.04	1594	35.658	0.91	1578	80.674
97	α-Humulene epoxide II	MS, KI	Os	1606	0.049	1612	36.333	-	-	-	0.12	1594	82.57
98	Epicubenol	MS, KI	Os	1627	0.051	1632	37.042	-	-	-	-	-	-
99	Methyl jasmonate	MS, KI	Es	1638	0.04	1652	37.775	-	-	-	-	-	-
100	α-Bisabolol	MS, KI, S	Os	1684	0.184	1687	39.092	-	-	-	0.45	1677	91.559
101	*Epi*-13-Manool	MS, KI	Od	2056	-	-	-	-	-	-	0.15	2056	115.15
Number of compounds					61			47			42		
Hydrocarbon monoterpenes (%)					53.35			4.27			13.98		
Oxygenated monoterpenes (%)					32.52			93.14			73.81		
Hydrocarbon sesquiterpenes (%)					12.39			1.31			5.73		
Oxygenated sesquiterpenes (%)					0.62			0.12			1.93		
Oxygenated diterpenes (%)					-			-			0.15		
Non-terpene derivatives (%)					-			0.36			0.54		
Total (%)					98.92			99.20			96.14		
Yield (%)					1.8			1.1			1.4		

Identif: identification. Cf: chemical family. KI lit: literature Kovats index according to the NIST20 database. REO: *S. rosmarinus* essential oil. MEO: *M. × piperita* essential oil. SEO: *S. officinalis* essential oil. %: areas according to TIC-MS chromatogram. KI ex: experimental Kovats index calculated against n-alkanes. RT: retention time. S: standard. MS: comparison of mass spectra. KI: comparison of experimental and literature Kovats indices. Ad: aldehyde. Al: alcohol. Td: tetrahydrofuran derivative. Hy: hydrocarbon. Bz: benzopyran. Es: ester. % ≥ 2: are presented in bold. -: absent.

**Table 2:** Cytotoxic properties (IC_50_ and selectivity index) and interaction effects of individual and combined essential oils of *Rosmarinus officinalis* (REO), *Mentha × piperita* (MEO), and *Salvia officinalis* (SEO) against normal (NHDF) and cancer (A549, MCF7, LoVo) cell lines.

Sample	Cell Line	IC_50_ ± SD (µg/mL)	SI	FIC_A_	FIC_B_	FIC Index	Interaction
REO	NHDF	537.7 ± 19.3	-	-			
	A549	32.3 ± 6.6	16.6				
	MCF7	13.7 ± 2.6	39.0				
	LoVo	968.53 ± 68.12	0.6				
MEO	NHDF	282.3 ± 19.9	-				
	A549	157.1 ± 6.6	1.8				
	MCF7	50.9 ± 15.7	5.5				
	LoVo	196.38 ± 4.5	1.4				
SEO	NHDF	325.5 ± 42.1	-				
	A549	80.5 ± 10.9	4.0				
	MCF7	9.7 ± 0.8	33.6				
	LoVo	829.31 ± 20.15	0.4				
REO + MEO	NHDF	410.5 ± 8.9	-	0.382	0.727	1.109	Indifferent
	A549	11.11 ± 1.26	36.9	0.172	0.035	0.207	Synergistic
	MCF7	85.06 ± 15.5	4.8	3.104	0.836	3.940	Indifferent
	LoVo	178.60 ± 10.80	2.3	0.092	0.455	0.547	Additive
REO + SEO	NHDF	278.3 ± 22.9	-	0.259	0.427	0.686	Additive
	A549	16.83 ± 4.32	16.5	0.261	0.105	0.365	Synergistic
	MCF7	27.42 ± 5.14	10.2	1.001	1.413	2.414	Indifferent
	LoVo	294.02 ± 18.60	0.1	0.152	0.177	0.329	Synergistic
MEO + SEO	NHDF	223.6 ± 19.9	-	0.396	0.343	0.740	Additive
	A549	84.46 ± 13.45	2.6	0.269	0.525	0.793	Additive
	MCF7	77.88 ± 4.15	2.9	0.765	4.014	4.779	Antagonistic
	LoVo	112.83 ± 4.10	2	0.287	0.068	0.355	Synergistic

REO: *S. rosmarinus* essential oil. MEO: *M. × piperita* essential oil. SEO: *S. officinalis* essential oil. NHDF: normal human dermal fibroblasts. A549: lung cancer cells. MCF7: breast cancer cells. LoVo: colorectal cancer IC_50_: essential oil concentration that reduces cell growth by 50%. SD: standard deviation. SI: selectivity index. FIC: fractional inhibitory concentration. FIC_A_ and FIC_B_ refer to the fractional inhibitory concentrations of each EO in the combination. -: not determined.

**Table 3:** log_10_ reduction factor and infectivity reduction (%) of *R. officinalis*, *M. × piperita*, and *S. officinalis* essential oils, and their 1:1 combination on AdV5 and HSV-1 after 15 and 60 min of contact time.

Samples	Virus							
	AdV5 15′		AdV5 60′		HSV-1 15′		HSV-1 60′	
	log_10_ Reduction	Infectivity Reduction	log_10_ Reduction	Infectivity Reduction	log_10_ Reduction	Infectivity Reduction	log_10_ Reduction	Infectivity Reduction
REO	>4 log_10_	>99.99%	>4 log_10_	>99.99%	>4 log_10_	>99.99%	>4 log_10_	>99.99%
MEO	4 log_10_	99.99%	>4 log_10_	>99.99%	4 log_10_	99.99%	>4 log_10_	>99.99%
SEO	>4 log_10_	>99.99%	4 log_10_	99.99%	4 log_10_	99.99%	>4 log_10_	>99.99%
REO + MEO	4 log_10_	99.99%	>4 log_10_	>99.99%	4 log_10_	99.99%	>4 log_10_	>99.99%
REO + SEO	4 log_10_	99.99%	>4 log_10_	>99.99%	>4 log_10_	>99.99%	>4 log_10_	>99.99%
MEO + SEO	1 log_10_	90%	4 log_10_	99.99%	4 log_10_	99.99%	>4 log_10_	>99.99%

REO: *S. rosmarinus* essential oil. MEO: *M. × piperita* essential oil. SEO: *S. officinalis* essential oil. AdV5: human adenovirus type 5, HSV-1: human herpes simplex virus 1, 15′: contact time of 15 min, 60′: contact time of 60 min.

**Table 4:** Free binding energies (kcal/mol) of the sixteen major compounds.

	Binding Energies (kcal/mol)					
Ligand	1Y8O	6NJS	3EYG	6CBZ	2KI5	2JV9
Co-Crystalized ligand	−7.3 ^a^	−8.9 ^b^	−6.4 ^c^	−6.9 ^d^	−6.1 ^e^	−5.8 ^f^
α-Pinene	−5.2	−4.7	−5.5	−6.1	−4.6	−5.2
β-Caryophyllene	−6.0	−5.5	−5.5	−5.1	−5.5	−7.0
β-Pinene	−5.5	−4.6	−4.7	−6.0	−4.7	−5.3
β-Thujone	−5.3	−5.4	−6.3	−6.2	−4.8	−5.3
Camphene	−4.9	−5.0	−5.3	−5.8	−4.6	−5.1
δ-Cadinene	−5.8	−5.5	−5.2	−5.3	−5.8	−7.2
Eucalyptol	−5.0	−5.1	−4.5	−6.2	−4.7	−5.4
γ-Terpineol, dihydro-	−5.1	−4.6	−4.7	−4.7	−4.5	−5.9
Isocaryophyllene	−6.4	−6.0	−5.5	−5.2	−5.8	−6.5
Isomenthone	−4.8	−4.8	−4.6	−5.7	−4.9	−5.1
Camphor	−5.0	−5.0	−5.5	−6.2	−5.0	−5.4
Limonene	−5.1	−4.8	−4.9	−4.8	−4.5	−5.2
Menthone	−4.7	−4.8	−5.6	−4.2	−4.4	−5.4
Menthyl acetate	−4.9	−5.5	−5.5	−4.6	−4.7	−5.5
*p*-Cymene	−5.1	−4.8	−4.3	−4.6	−4.2	−5.6
α-Thujone	−5.0	−4.9	−5.0	−6.5	−6.5	−5.3

1Y8O: PDB ID of pyruvate dehydrogenase kinase 3. 6NJS: PDB ID of signal transducer and activator of transcription 3. 3EYG: PDB ID of tyrosine-protein kinase. 6CBZ: PDB ID of estrogen receptor alpha ligand binding domain. 2KI5: PDB ID of HSV-1 thymidine kinase. 2JV9: PDB ID of HSV-1 DNA polymerase. ^a^: adenosine diphosphate. ^b^: compound SD36. ^c^: Tofacitinib. ^d^: Estradiol. ^e^: Acyclovir. ^f^: 4-Oxo-dihydroquinoline-3-carboxamide.

**Table 5:** Physicochemical properties, pharmacokinetics, drug-likeness, and lipophilicity of the selected compounds.

Molecule	1	2	3	4	5	6	7	8	9	10	11	12	13	14	**15**	**16**
Physicochemical properties																
Molecular formula	C_10_H_16_	C_15_H_24_	C_15_H_24_	C_10_H_16_	C_10_H_16_	C_10_H_18_O	C_15_H_24_	C_10_H_18_O	C_10_H_16_O	C_10_H_18_O	C_10_H_16_O	C_10_H_16_O	C_10_H_16_	C_10_H_18_O	C_12_H_22_O_2_	C_10_H_14_
Molecular weight (g/mol)	136.23	204.35	204.35	136.23	136.23	154.25	204.35	154.25	152.23	154.25	152.23	152.23	136.23	154.25	198.30	134.22
Rotatable bonds	0	1	0	0	0	0	0	1	1	0	1	0	1	1	3	1
H-bond acceptors	0	0	0	0	0	1	0	1	1	1	1	1	0	1	2	0
H-bond donors	0	0	0	0	0	1	0	0	0	0	0	0	0	0	0	0
TPSA (å^2^)	0	0	0	0	0	20.23	0	17.07	17.07	9.23	17.07	17.07	0	17.07	26.3	0
Lipophilicity																
Consensus Log P	3.44	4.14	4.24	3.42	3.43	2.41	4.24	2.6	2.35	2.67	2.35	2.37	3.37	2.61	3	3.5
Absorption																
GI absorption	Low	Low	Low	Low	Low	High	Low	High	High	High	High	High	Low	High	High	Low
Skin permeability ^a^	−3.95	−4.85	−4.44	−4.18	−4.13	−5.73	−4.44	−5.08	−5.62	−5.3	−5.62	−5.67	−3.89	−5.08	−4.67	−4.21
Distribution																
BBB permeant ^b^	Yes	No	No	Yes	Yes	Yes	No	Yes	Yes	Yes	Yes	Yes	Yes	Yes	Yes	Yes
Pgb substrate	No	No	No	No	No	No	No	No	No	No	No	No	No	No	No	No
Metabolism																
CYP1A2 inhibitor	No	No	No	No	No	No	No	No	No	No	No	No	No	No	No	No
CYP2C19 inhibitor	No	Yes	Yes	No	No	No	Yes	No	No	No	No	No	No	No	No	No
CYP2C9 inhibitor	Yes	Yes	Yes	Yes	Yes	No	Yes	No	No	No	No	No	Yes	No	Yes	No
CYP2D6 inhibitor	No	No	No	No	No	No	No	No	No	No	No	No	No	No	No	Yes
CYP3A4 inhibitor	No	No	No	No	No	No	No	No	No	No	No	No	No	No	No	No
Drug-likeness																
Bioavailability score	0.55	0.55	0.55	0.55	0.55	0.55	0.55	0.55	0.55	0.55	0.55	0.55	0.55	0.55	0.55	0.55
Excretion																
Total clearance ^c^	0.043	1.182	1.088	0.03	0.049	1.122	1.088	0.244	0.135	1.009	0.135	0.109	0.213	0.244	1.207	0.239
Renal OCT2 substrate	No	No	No	No	No	No	No	No	No	No	No	No	No	No	No	No
Toxicity																
AMES toxicity	No	No	No	No	No	No	No	No	No	No	No	No	No	No	No	No
Hepatotoxicity	No	No	No	No	No	No	No	No	No	No	No	No	No	No	No	No

TPSA: Topological polar surface area, BBB: Blood–brain barrier, GI: Gastrointestinal, P-gp: P-glycoprotein, ^a^ (log Kp, cm/s), ^b^ (C.brain/C.blood), CYP: Cytochrome P450 enzymes, ^c^ log ml/min/kg. OCT2: Organic cation transporter 2. AMES: Bacterial Reverse Mutation Assay, 1: α-Pinene, 2: δ-Cadinene, 3: β-Caryophyllene, 4: β-Pinene, 5: Camphene, 6: γ-Terpineol, dihydro-, 7: Isocaryophyllene, 8: Isomenthone, 9: β-Thujone, 10: Eucalyptol, 11: α-Thujone, 12: Camphor, 13: Limonene, 14: Menthone, 15: Menthyl acetate, 16: *p*-Cymene.

**Table 6:** The main characteristics of species used in the study.

Features	Plant Species		
	*Salvia rosmarinus*	*Mentha piperita*	*Salvia officinalis*
Vernacular name	Rosemary	Peppermint	Common Sage
Geographic origin	Chelia Forest, Khenchela, Algeria	Bouraoui Belhadef Forest, Jijel, Algeria	Aïn Beïda, Oum El Bouaghi, Algeria
Elevation (m)	1320	612	924
Longitude	06°48′40″ E	06°06′04″ E	07°20′14″ E
Latitude	35°24′34″ N	36°41′43″ N	35°47′20″ N
Voucher No.	2023AKSR	2023AKMP	2023AKSO
Harvest period	June 2023	October 2023	October 2023

## Data Availability

The data that support the findings of this study are available from the corresponding author on reasonable request.

## Abbreviations

The following abbreviations are used in this manuscript:

A549	Human Lung Carcinoma Cell Line
ADMET	Absorption, Distribution, Metabolism, Excretion, and Toxicity
AdV5	Human Adenovirus Type 5
AMES	Bacterial Reverse Mutation Assay
ATCC	American Type Culture Collection
BBB	Blood–Brain Barrier
C	Control Growth
CC	Cell Control
CG%	Cell Growth Percentage
CPE	Cytopathic Effect
CYP	Cytochrome P450 enzymes
DMEM	Dulbecco’s Modified Eagle Medium
DNA	Deoxyribonucleic Acid
DPBS	Dulbecco’s Phosphate-Buffered Saline
ECACC	European Collection of Authenticated Cell Cultures
EMEM	Eagle’s Minimum Essential Medium
EO(s)	Essential Oil(s)
ERαLBD	Estrogen Receptor Alpha Ligand Binding Domain
FBS	Fetal Bovine Serum
FFNSC	Flavors and Fragrances of Natural and Synthetic Compounds
FIC	Fractional Inhibitory Concentration
GC-MS	Gas Chromatography-Mass Spectroscopy
GI	Gastrointestinal
HeLa	Human Cervix Carcinoma Cell Line
HSV-1	Human Herpes Simplex Virus Type 1
IC_50_	50% Inhibitory Concentration
JAK1	Tyrosine-Protein Kinase
KI	Kovats Retention Index
LGA	Lamarckian Genetic Algorithm
Log Kp	Logarithm of Skin Permeability Coefficient
LoVo	Human Colorectal Cancer Cell line
MCF7	Human Breast Cancer Cell Line
MD	Molecular Docking
MEO	Essential Oil of *Mentha × piperita* L.
MNTC	Maximum Non-Toxic Concentration
NCI60	National Cancer Institute 60 Human Tumor Cell Line Panel
NHDF	Normal Human Dermal Fibroblasts
NIST	National Institute of Standards and Technology
OCT2	Organic Cation Transporter 2
PBS	Phosphate-Buffered Saline
PDB	Protein Data Bank
PDK3	Pyruvate Dehydrogenase Kinase 3
P-gp	P-Glycoprotein
REO	Essential Oil of *Salvia rosmarinus* Spenn. (REO),
RT	Retention Time
SD	Standard Deviation
SEO	Essential Oil of *Salvia officinalis* L.
SI	Selectivity Index
SRB	Sulforhodamine B
STAT3	Signal Transducer and Activator of Transcription 3
TCA	Trichloroacetic Acid Solution
TCID_50_	Tissue Culture Infective Dose 50%
Ti	Treated Cells (at given concentration)
TPSA	Topological Polar Surface Area
Tz	Time Zero
VC	Virus Control
